# Strophanthidin Induces Apoptosis of Human Lung Adenocarcinoma Cells by Promoting TRAIL-DR5 Signaling

**DOI:** 10.3390/molecules29040877

**Published:** 2024-02-16

**Authors:** Xiao Tian, Liangzhen Gu, Fangang Zeng, Xingkai Liu, Yang Zhou, Yang Dou, Juanjuan Han, Yao Zhao, Yanyan Zhang, Qun Luo, Fuyi Wang

**Affiliations:** 1 College of Traditional Chinese Medicine, Shandong University of Traditional Chinese Medicine, Jinan 250355, China; tianxiaodeyouxiang@163.com; 2 Beijing National Laboratory for Molecular Sciences, CAS Research/Education Center for Excellence in Molecular Sciences, National Centre for Mass Spectrometry in Beijing, CAS Key Laboratory of Analytical Chemistry for Living Biosystems, Institute of Chemistry, Chinese Academy of Sciences, Beijing 100190, China; guliangzhen@iccas.ac.cn (L.G.); liuxingkai@iccas.ac.cn (X.L.); zhouyang14@mails.ucas.ac.cn (Y.Z.); douyang18@mails.ucas.ac.cn (Y.D.); hjuan@iccas.ac.cn (J.H.); yaozhao@iccas.ac.cn (Y.Z.); zhangyy0816@iccas.ac.cn (Y.Z.); 3 University of Chinese Academy of Sciences, Beijing 100049, China; 4 School of Environment of Natural Resources, Remin University of China, Beijing 100875, China; zengfg@ruc.edu.cn

**Keywords:** strophanthidin, anticancer agent, apoptosis, Trail-DR4/5 signaling, mass spectrometry, quantitative proteomics, bioinformatics

## Abstract

Strophanthidin (SPTD), one of the cardiac glycosides, is refined from traditional Chinese medicines such as *Semen Lepidii* and *Antiaris toxicaria*, and was initially used for the treatment of heart failure disease in clinic. Recently, SPTD has been shown to be a potential anticancer agent, but the underlying mechanism of action is poorly understood. Herein, we explored the molecular mechanism by which SPTD exerts anticancer effects in A549 human lung adenocarcinoma cells by means of mass spectrometry-based quantitative proteomics in combination with bioinformatics analysis. We revealed that SPTD promoted the expression of tumor necrosis factor (TNF)-related apoptosis-inducing ligand receptor 2 (TRAIL-R2, or DR5) in A549 cells to activate caspase 3/6/8, in particular caspase 3. Consequently, the activated caspases elevated the expression level of apoptotic chromatin condensation inducer in the nucleus (ACIN1) and prelamin-A/C (LMNA), ultimately inducing apoptosis via cooperation with the SPTD-induced overexpressed barrier-to-autointegration factor 1 (Banf1). Moreover, the SPTD-induced DEPs interacted with each other to downregulate the p38 MAPK/ERK signaling, contributing to the SPTD inhibition of the growth of A549 cells. Additionally, the downregulation of collagen COL1A5 by SPTD was another anticancer benefit of SPTD through the modulation of the cell microenvironment.

## 1. Introduction

Natural products have been a valuable source of modern drug discovery for a long time, especially in the treatment of tumors and malaria [1,2,3,4]. Paclitaxel was the first chemotherapeutic drug extracted from natural plants, e.g., the bark and leaves of yew trees. Paclitaxel has been one of the first-line chemotherapeutic drugs for the clinical treatment of breast cancer, ovarian cancer, lung cancer and some head and neck cancers during the past five decades [2,5]. Vinblastine, as well as its analog, vincristine, are both natural products refined from the dried leaves of *Catharanthus roseus*, and have been clinically used along with other chemotherapeutic drugs for the treatment of many types of cancers, including lymphoma, testicular cancer, ovarian cancer, breast cancer, bladder cancer, and lung cancer [6]. Vinblastine inhibits leukopoiesis and is the mainstay of treatment for pediatric lymphocytic leukemia and non-Hodgkin lymphoma [6]. Being the milestone of research and development in traditional Chinese medicine, artemisinin, a sesquiterpene lactone with unique chemical structures derived from *Artemisia annua* L., has gained wide acceptance as the first-line antimalarial drug [7].

Cardiac glycosides (CGs), also known as cardiosteroids, are natural products with a steroid-like structure and unsaturated lactone rings. CGs are mainly derived from a variety of plants and amphibians, including digitalis, oleander, and Brazilian toad [8], and are clinically used widely in the treatment of heart failure and arrhythmias [8]. Being one of the CG family, strophanthidin (SPTD) is structurally a cardanolide with only one aglycone and no sugar unit. It is refined from traditional Chinese medicines such as *Semen Lepidii* and *Antiaris toxicaria*. Clinical and laboratory studies have indicated that SPTD was efficacious in alleviating heart failure diseases [9,10]. Meanwhile, SPTD has been shown to induce apoptosis and cause cell cycle arrest of cancer cells [11,12]. Reddy et al. demonstrated that SPTD downregulated the expression of protein kinases, such as PI3K, MEK3, AKT and GSK3-α to regulate MAPK, PI1K/AKT/mTOR and Wnt/β-catenin signaling [12]. However, to the best of our knowledge, few studies have been performed to investigate, on a proteome scale, the underlaying mechanism of SPTD-induced apoptosis and cell death.

Proteomics on a proteome-wide scale explores the expression, functions, posttranslational modifications and interactions of all proteins in a type of cells and/or biological tissues. Over the past two decades, due to advances in mass spectrometry instrumentations and data analysis techniques, mass spectrometry (MS)-based proteomics have emerged as a powerful tool for identifying and validating drug therapeutic targets, providing unique insights into better understanding the mechanism of action, and for identifying safety hazards of drug candidates and clinically used drugs, including natural products [13,14,15]. CCCP strategy involves the rational design of target affinity probes by using modified drug molecules to separate and enrich the target proteins of the drugs for subsequent mass spectrometry analysis [16]. For example, Wang et al. developed a compound-centric chemical proteomics (CCCP) method to delineate the mechanism of action of the well-recognized anti-malarial drug artemisinin. They applied an alkyne-tagged artemisinin analogue tethered with a biotin group to label and subsequently fish the artemisinin binding protein targets via biotin-avidin interaction for MS identification. They identified 124 artemisinin targets which disrupted the parasite’s haem biosynthesis pathway and kill parasite [17]. Meanwhile, Ismail et al. developed a similar artemisinin (ART) activity-based protein profiling probe to label the molecular targets of ART by alkylating ART protein targets in plasmodium falciparum 3D7 parasites for subsequent MS analysis [18]. They demonstrated that ART targets are mainly involved in the glycolytic, hemoglobin degradation, protein synthesis and antioxidant defense pathways, which are all associated with parasite survival [18]. Recently, we assembled a baicalin-functionalized magnetic probe to capture the protein targets of baicalin from cell lysates. The subsequent MS quantification identified 14 baicalin targets, most of which are ATP-binding and/or ATPase activity proteins, such as CKB, HSP86 and ATPSF1β [19].

Alternatively, mechanism centric proteomics (MCP) methods can be performed to investigate the protein targets and mechanism of action of candidate drugs and druggable compounds. Recently, we applied MS quantification coupled to tandem-mass tag (TMT) labeling to decipher the gene targets of a G-quadruplex (G4) DNA inducer/stabilizer, pyridostatin (PDS). We found that PDS targeted the *SUB1* gene and dramatically reduced the expression of its encoded protein, human positive cofactor (PC4), which in turn promoted the cytotoxicity of a transplatinum complex (trans-[PtCl_2_(NH_3_)(thiazole)]) against HeLa cells via the reduction of the repair of platinate-crosslinked DNA lesions, mediated by PC4 [20]. Combined with the bioinformatics analysis, we also used a similar MCP method to investigate the mechanism of action of a ruthenium polypyridine complex, [Ru(dppa)_2_(pytp)](PF_6_)_2_, where dppa = 4,7-diphenyl-1,10-phenanthroline and pytp = 4′-pyrene-2,2′:6′,2″-terpyridine as a photoactivatable anticancer agent. We revealed that the ruthenium complexes upon light irradiation at 470 nm produced reactive oxygen species (ROS) to activate oxidative phosphorylation signaling and generated ROS and pyrene derivatives to damage DNA, which synergistically led to oncotic necrosis and apoptosis of the A549 human lung adenocarcinoma cells [21]. In order to delineate the molecular mechanism of action of SPTD against cancer cells, in the present work, we applied MS-based MCP methods to profile the expression of proteins in A549 cells. Significantly, we demonstrated that SPTD stimulated the expression of the death receptor DR5, activating caspase 3/6/8 and inducing the apoptosis of A549 cells.

## 2. Results

### 2.1. Cytotoxicity of Strophanthidin

**Figure 1:**
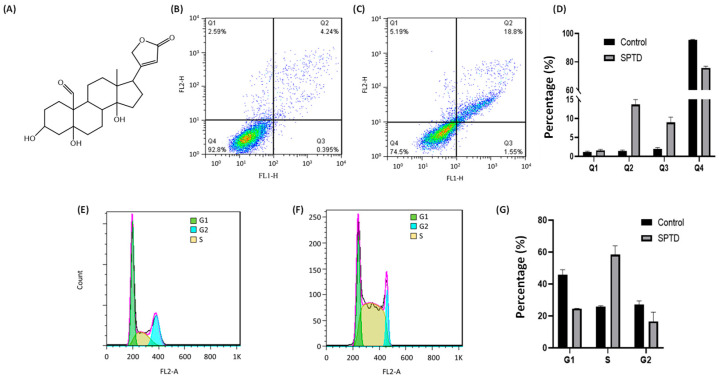
(**A**) Chemical structure of strophanthidin (SPTD). (**B**,**C**) Flow cytometric quantification of viability (Q1), early-stage apoptosis (Q2), late-stage apoptosis (Q3) and necrosis (Q4) of A549 cells without ((**B**), control) and with ((**C**), SPTD) treatment of 1.0 μM SPTD at 37 °C for 24 h. (**D**) Percentage of cells at various states in the control and SPTD groups of A549 cells (n = 3). (**E**,**F**) Cell cycle profiles of A549 cells without (**E**) and with (**F**) treatment of 1.0 μM SPTD at 37 °C for 24 h. (**G**) Histograms of population distribution in different cell cycles of A549 cells without (control) and with (SPTD) treatment of 1.0 μM SPTD at 37 °C for 24 h.

To verify the anticancer activity of strophanthidin (SPTD) (Figure 1A), we firstly measured the half-maximum inhibitory concentration (IC_50_) of SPTD towards the A549 lung adenocarcinoma cells, compared with human embryonic kidney HEK293T cells as a normal cell line control. The results showed that the proliferation of both A549 and HEK293T cells were inhibited by SPTD in a dose-dependent manner (Figure S1 in the Supplementary Materials). However, the IC_50_ of SPTD towards HEK293T cells (3.02 ± 0.21 μM) was about six times that of the IC_50_ against A549 cells (0.51 ± 0.12 μM), indicating that SPTD is much less cytotoxic to normal HEK293T cells. Compared to the first-line chemotherapeutic drug cisplatin, of which the IC_50_ towards A549 cells was 10.4 ± 1.0 μM for 48 h of treatment [22], the results indicate that SPTD is highly cytotoxic to A549 cancer cells.

### 2.2. Cellular Phenotype Characterization of A549 Cells Treated with SPTD

Cell death induced by anticancer drugs mainly occurs in apoptosis and necrosis. To further characterize the cytotoxicity of SPTD to A549 cells, we applied flow cytometry to investigate the death pathways of A549 cells treated with and without SPTD treatment. As shown in Figure 1B,D, without SPTD exposure, 1.17%, 1.38% and 2.18% of A549 cells underwent early-stage apoptosis, late-stage apoptosis and necrosis, respectively. In comparison, 1.80%, 13.8% and 7.55% of A549 cells were induced to early-stage apoptosis, late-stage apoptosis and necrosis, respectively, with a treatment of 1.0 μM SPTD (Figure 1C,D). This suggests that SPTD induces the death of A549 cells mainly via late-stage apoptosis.

Next, we investigated the effect of SPTD on the cell cycle of A549 cells. We found that compared with the control group of cells without SPTD treatment, the populations of G1 and G2 phases of A549 cells in the SPTD treatment group were decreased from 45.81% to 24.64%, and from 27.24% to 16.54%, respectively. In contrast, the population of the S phase of A549 cells increased from 25.91% to 58.41% upon exposure to 1 μM of SPTD for 24 h (Figure 1F,G). These indicate that SPTD treatment leads to the arrest of A549 cells at S phase.

### 2.3. Quantitative Proteomics Analysis

**Figure 2:**
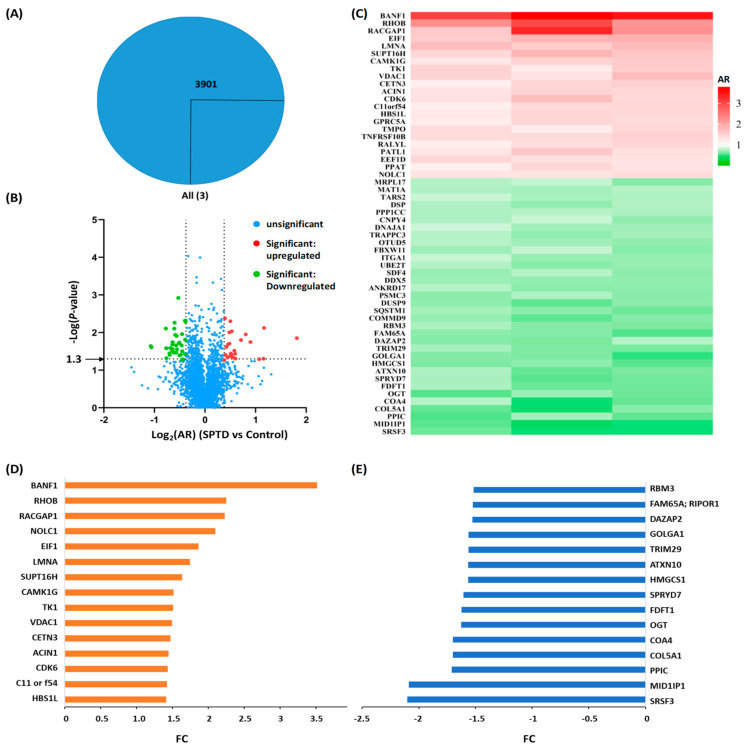
Quantitative proteomics analysis. (**A**) Venn diagram of the numbers of the proteins commonly expressed in A549 cells without (control) and with SPTD (1.0 μM) treatment. (**B**) The volcanic map of the proteins identified in both control and treated groups of A549 cells with various abundance ratios (ARs, SPTD vs. control) and *p*-values. Blue dots refer to proteins with statistically insignificant change in expression level between the two groups of cells, while red and green ones refer to proteins with statistically significant change in expression level between the two groups of cells with *p* ≤ 0.05 (−lg *p* ≥ 1.3) and log_2_AR ≥ 0.38, and with *p* ≤ 0.05 (−lg *p* ≥ 1.3) and log_2_AR ≤ −0.38. (**C**) The heat-map of the differentially expressed proteins (DEPs) with *p* ≤ 0.05 and |log_2_AR| ≥ 0.38 subjected to SPTD treatment. (**D**,**E**) The fold-change (FC) of the 15 top upregulated DEPs (orange) (**D**) and the top downregulated DEPs (blue) (**E**).

To elucidate the molecular mechanism of action of the SPTD-induced apoptosis of A549 cells, we applied quantitative proteomics analysis based on mass spectrometry (MS) coupled to the tandem-mass-tag (TMT) labeling technique to profile the protein expression of A549 cells upon SPTD treatment. By analyzing three independent biological replicates of A549 cells without and with treatment of 1 μM SPTD at 37 °C for 24 h, we identified 3901 proteins which were commonly expressed in both the SPTD-untreated group and SPTD-treated group of A549 cells (Figure 2A) by matching the MS/MS data to the Protein Discovery (PD) library. Among the 3901 proteins were 312 proteins that were statistically reliable with a *p*-value ≤0.05 (log *p* ≤ 1.3, Table S1), of which 35 proteins were significantly downregulated (green dots, *p* < 0.05, lg_2_(AR) ≤ −1.3), where AR represents the ratio of the abundance of a protein expressed in the untreated group of cells to the same protein in the SPTD-treated group of cells, and 24 proteins were significantly upregulated (red dots, *p* < 0.05, log_2_(AR) ≥ 1.3) (Figure 2B). The abundance ratios measured by the MS analysis of the three biological replicates of the 59 differentially expressed proteins (DEPs) between the control group and the SPTD-treated group were presented as a heatmap (Figure 2C), which indicates the good repeatability of three biological replicates.

To straightforwardly indicate the changes in the expression level of proteins between the cells of SPTD-treated and untreated groups, we introduced fold-change (FC) to represent the change in the expression level of all DEPs. When AR > 1, FC is equal to AR, indicating the protein was upregulated; when AR < 1, FC is equal to the negative reciprocal of AR, indicating the protein was downregulated in the SPTD-treated group, compared to the same protein expressed in the control group. Accordingly, the FCs of the 15 most upregulated and the 15 most downregulated DEPs are shown in Figure 2D,E, the FCs of the rest of DEPs with |FC| > 1.3 are shown in Figure S2. It is notable that SPTD treatment elevated the expression of barrier-to-autointegration factor 1 by 3.52-fold, while decreasing the expression of serine/arginine-rich splicing factor 3 by 2.10. The barrier-to-autointegration factor 1 (Banf1) is one of the nuclear envelope proteins, and has been reported to guide membranes to form a single nucleus by bridging distant DNA sites during mitosis, playing a key role in nuclear assembly [23]. Banf1 was also reported to control the DNA damage response to oxidative stress through the regulation of poly [ADP-ribose] polymerase 1 (PARP1) [24]. With regard to this, the significant upregulation of Banf1 by SPTD may contribute to the antiproliferative activity of SPTD on A549 cells. However, the underlying mechanism requires further study. The most downregulated protein, serine/arginine-rich splicing factor 3 (SRSF3), by SPTD is a splicing factor and a nuclear export adaptor protein that specifically promotes exon-inclusion during alternative splicing by interacting with YTHDC1 which serves as the reader of N^6^-methyladenosine (m^6^A) [25,26,27]. Hence, the SPTD-induced downregulation of SRSF3 may contribute directly to the inhibition of SPTD on the growth of A549 cells by retarding the mRNA splicing.

### 2.4. Bioinformatics Analysis

**Figure 3:**
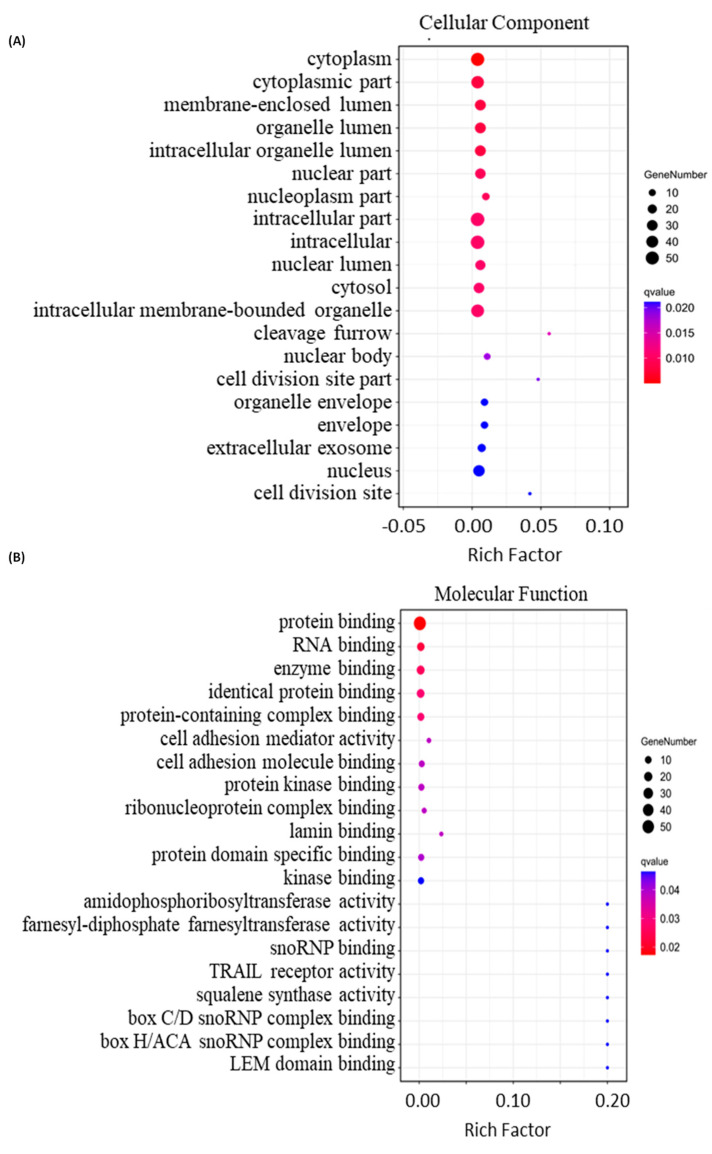
Bioinformatics analysis of the differentially expressed proteins (DEPs) with |FC| ≥ 1.3 identified in A549 cells treated with 1.0 μM of SPTD. (**A**) Top 20 cellular components, (**B**) top 20 molecular functions.

**Figure 4:**
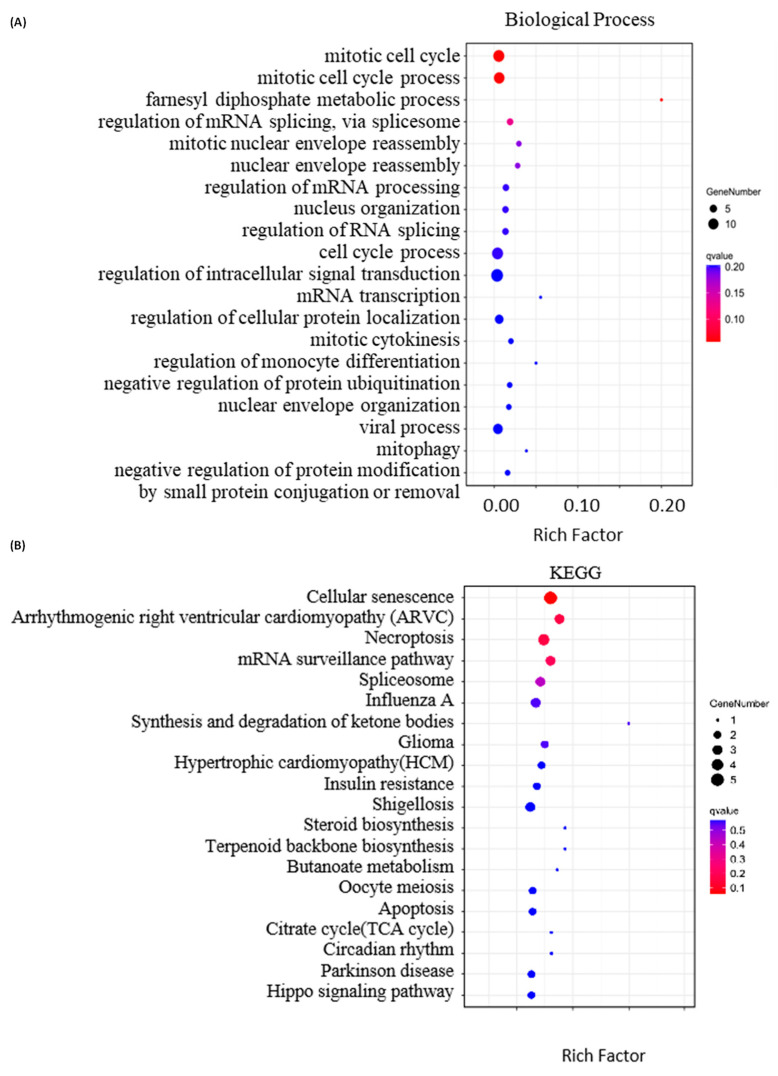
Bioinformatics analysis of the differentially expressed proteins (DEPs) with |FC| ≥ 1.3 identified in A549 cells treated with 1.0 μM of SPTD. (**A**) Top 20 involved biological processes and (**B**) top 20 associated KEGG pathways of the DEPs.

Next, we performed bioinformatics analysis on the DEPs. Firstly, we utilized OmicShare for Gene Ontology analysis to annotate the cellular components, molecular functions and involved biological processes of the DEPs with |FC| ≥ 1.3 (Table S2). The results indicate that the 59 DEPs are mainly located in the cytoplasm, membrane lumen and organelle lumens (Figure 3A). The DEPs bind to proteins, RNA and enzymes (Figure 3B), and are mainly involved in the mitotic cell cycle, farnesyl diphosphate metabolism and regulation of mRNA splicing via spliceosomes (Figure 4A). Notably, the DEPs also show protein kinase binding activity (Figure 3B), implicated in the regulation of protein phosphorylation, which is in line with the previous report that peruvoside, a mono-glycosylated analog of SPTD, inhibits the MAPK, Wnt/β-catenin and PI3K/AKT/mTOR signaling pathways to induce apoptosis and autophagy [28]. Significantly, the DEPs have shown TRAIL receptor activity (Figure 3B). TRAIL is an apoptosis inducer, which has at least four cell membrane receptors, including the death receptor DR5 [29,30]. Herein, our quantitative proteomics analysis revealed that SPTD treatment elevated the expression of the gene *TNFRSF10B,* which expresses the tumor necrosis factor receptor superfamily member 10 (TNFRSF10B, also known as TRAIL-R2 or DR5), by 1.4-fold. These results indicate that SPTD triggered apoptosis via the promotion of the expression of DR5.

In accord with the annotation of GO analysis, KEGG (Kyoto Encyclopedia of Genes and Genomes) annotation indicates that the DEPs described above are highly associated with cellular senescence, necroptosis and mRNA surveillance (Figure 4B). Moreover, KEGG annotation revealed that SPTD-induced DEPs are closely associated with arrhythmogenic right ventricular cardiomyopathy (ARVC), hypertrophic cardiomyopathy (HCM) and circadian rhythm. This supports the pharmacological functions of SPTD as one of the cardiac glycosides.

**Figure 5:**
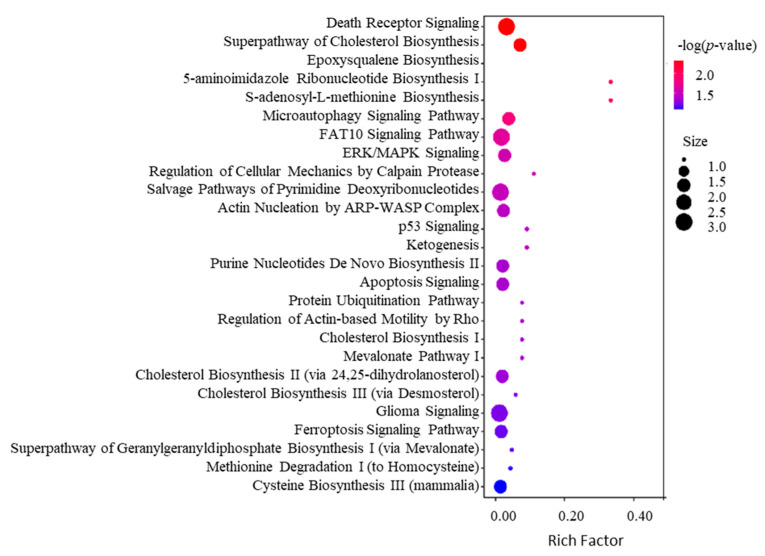
Top 20 associated canonical pathways of the differentially expressed proteins (DEPs) with |FC| ≥ 1.3 identified in A549 cells treated with 1.0 μM of SPTD enriched by IPA.

**Figure 6:**
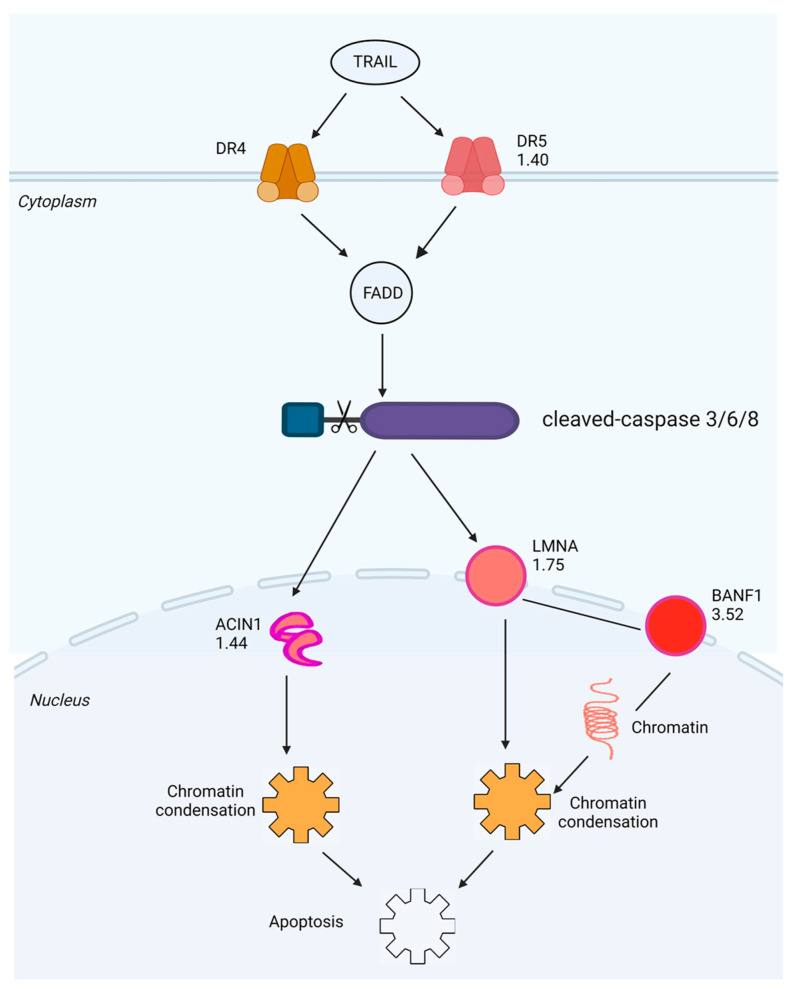
Schematic diagram generated by IPA of the death receptor signaling pathway with which the DEPs with |FC| ≥ 1.3 identified in A549 cells exposed to 1.0 μM of SPTD are highly associated with. The red color represents a protein (or complex) that is upregulated, green represents downregulation and the fold-change value is noted below the gene name.

To further decipher the mechanism of action of SPTD-induced apoptosis and cell death, we enriched the associated canonical pathways of the DEPs with |FC| ≥ 1.3 using the Ingenuity Pathway Analysis (IPA) program. The IPA enrichment (Figure 5) showed that the top five most associated core pathways of the DEPs are the death receptor signaling (Figure 6), super-pathway of cholesterol biosynthesis (Figure S3), epoxy-squalene biosynthesis (Figure S4), 5-aminoimidazole ribonucleotide biosynthesis I (Figure S5) and S-adenosyl-L-methionine biosynthesis (Figure S6) pathways. These indicate that apart from activating the TRAIL-DR5 signaling to induce the apoptosis of A549 cells (Figure 5), SPTD is also involved in the regulation of cholesterol, ribonucleotides and amino-acids biosynthesis processes, contributing to the inhibition of cell growth. Moreover, SPTD was demonstrated to be closely associated with the micro-autophagy signaling pathway (Figure S7) and the FAT10 signaling pathway (Figure S8), in both of which the 26S proteasome regulatory subunit 6A and sequestosome-1 encoded by *PSMC3* and *SASTM1*, respectively, were downregulated by SPTD by 1.48-fold and 1.49-fold. This implies that both of the signaling pathways were retarded by SPTD. However, the underlying functions of this regulation of anticancer activity require further investigation. Interestingly, the IPA enrichment showed that the SPTD-induced DEPs are involved in the regulation of the ERK/MAPK signaling pathway (Figure S9), again implying that SPTD may inhibit the growth of A549 cells by regulating protein phosphorylation through ERK/MARK signaling (*vide supra*).

**Figure 7:**
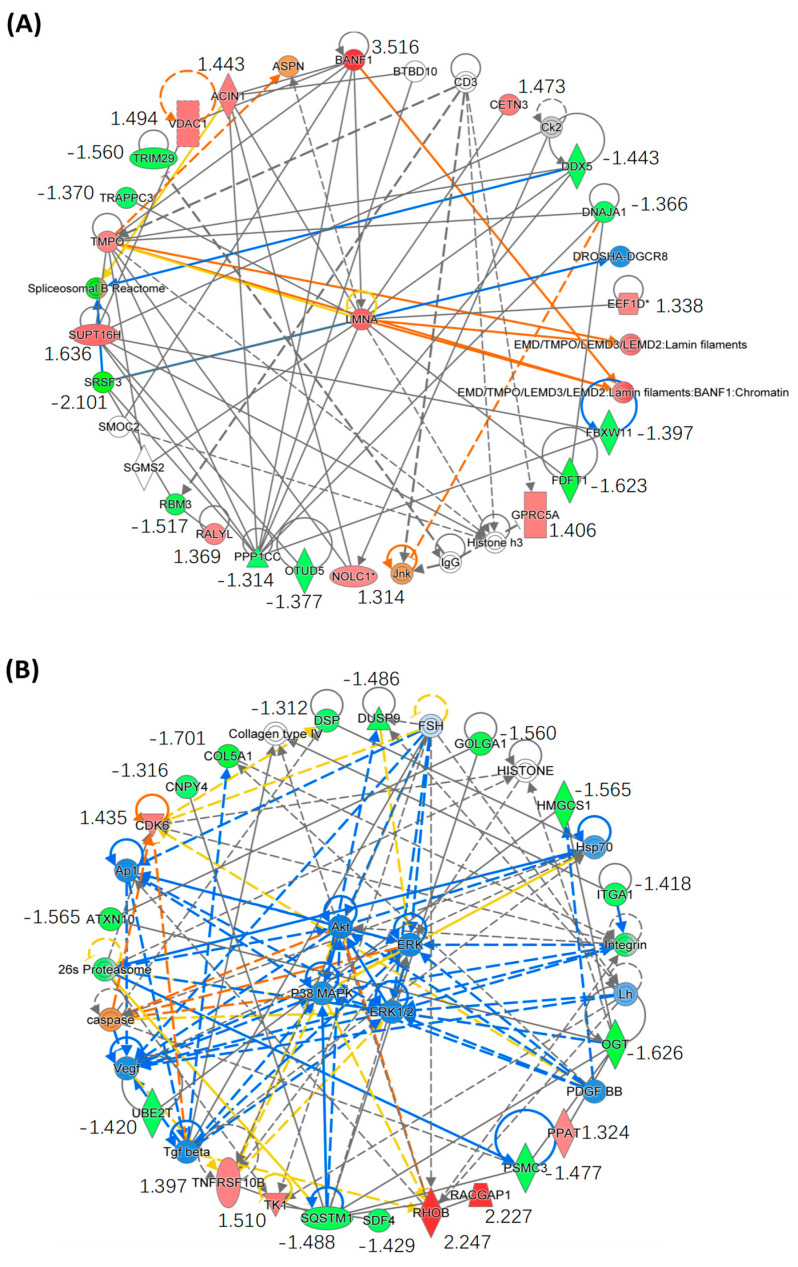
Enrichment of the core protein–protein interaction (PPI) networks of DEPs with |FC| ≥ 1.3 identified in A549 cells exposed to SPTD. (**A**) This PPI network indicates that all of the DEPs involved interact with each other via the core protein LMNA. (**B**) This PPI network indicates that all DEPs involved downregulate p38 MARK/ART/ERK signaling via interaction with VEGF, PDGF and TGFβ. The red color refers to upregulation of the genes, and green to downregulation, with fold-change value listed below the gene names. The orange color indicates that the gene is predicted by IPA to be upregulated or activated, blue indicates it is downregulated and grey indicates there is no prediction available. The solid line indicates direct interaction between two proteins and the dot line indicates indirect interaction. The asterisk (*) after a gene (or protein) name indicates that multiple identifiers in the dataset file map to a single gene (or protein) in the Global Molecular Network.

We further performed bioinformatics analysis by using IPA to generate the protein-protein interaction (PPI) networks of the DEPs described above. Interestingly, IPA enriched two PPI networks with which the DEPs are involved, in one of which all DEPs directly or indirectly interact with each other through the core protein LMNA (Figure 7A). The *LMNA* gene encodes two nuclear envelope proteins, Lamins A and C, which are present in equal amounts in the nuclear lamina and play an important role in DNA repair, nuclear assembly and chromatin organization [31]. As shown in Figure S2, our study shows that SPTD elevated the expression of DR5 (TNFRSF10B), activating TRAIL/DR5 signaling, which in turn activated the caspase cascade to increase the expression of LMNA (FC = 1.75), ultimately inducing apoptosis (Figure 6). In another IPA-enriched PPI network, the DEPs are demonstrated to interact with each other to downregulate the core kinases, including p38 MAPK, Akt and ERK 1/2 (Figure 7B).

### 2.5. Investigation of SPTD-Induced Apoptosis via Death Receptor Signaling by Fluorescence Imaging

**Figure 8:**
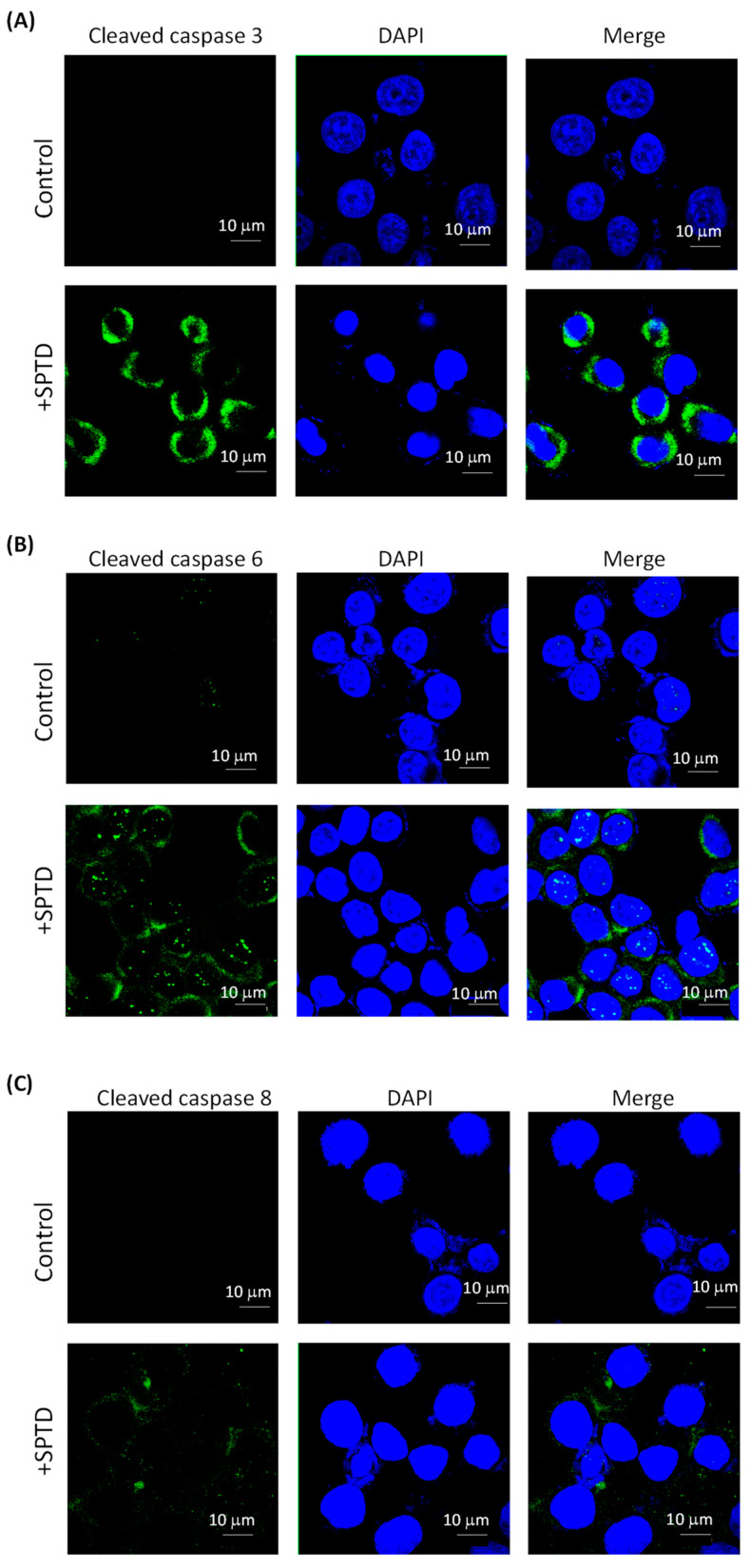
Immunofluorescence images of cleaved/activated caspase 3 (**A**), 6 (**B**) and 8 (**C**) in A549 without (control) and with treatment of 1.0 μM SPTD for 3 h at 37 °C. Green fluorescence represents the target protein (λ_ex_ = 488 nm, λ_em_ = 525 nm) and blue fluorescence represents the nucleus stained by DAPI (λ_ex_ = 360 nm, λ_em_ = 460 nm).

Both GO and IPA annotations revealed that SPTD targets the TRAIL/DR5 signaling pathway, which is a core pathway triggering cell death [29]. The apoptosis-inducing ligand, TRAIL, forms a complex with the death receptors DR5 or DR4 [30] to trigger receptor trimerization and generate homophilic interaction surfaces for death domain-containing adaptor proteins. The adaptor proteins in turn activate the caspase cascade by cleaving caspases 3, 6 and/or 8 [29]. Because the quantitative proteomics analysis cannot detect whether the caspases were cleaved or not, we applied immunofluorescence imaging to detect cleaved caspase proteins. The results showed that the level of cleaved caspase 3/6/8 in the SPTD-treated cells was significantly higher than that in the control group (Figure 8). Furthermore, the level of cleaved caspase 3 was higher than those of caspase 6 and 8, indicating that SPTD induces apoptosis mainly via the activation of caspase 3. Moreover, our results have also shown that the activation (or cleavage) of caspase 3 reached a maximum when the A549 cells were incubated with SPTD for 3 h, and then decreased dramatically, whereas the level of cleaved caspase 6/8 had no significant changes over the period of 3–12 h (Figures S10–S12). These results indicate that pharmacological efficiency may reach a maximum at ca. 3 h.

## 3. Discussion

As mentioned earlier, acting as a type of Na^+^/K^+^-ATPase inhibitor, cardiac glycosides (CGs) have been clinically used for the treatment of heart failure and atrial arrhythmias for more than 200 years [32,33]. Interestingly, since 1967, increased in vivo and in vitro evidence has demonstrated the anticancer properties of CGs [33,34,35,36]. Stenkvist et al. performed a long-term (22.3 years) follow-up study of 175 patients with breast carcinoma and found that the patients who received digitalis treatment had a lower death rate (6%) from breast carcinoma, compared with patients without digitalis treatment (34%) [36]. Afterwards, more clinical reports confirmed that CGs have significant therapeutic effects on breast cancer, prostate cancer, lung cancer, kidney cancer, pancreatic cancer, melanoma, leukemia, neuroblastoma and myeloma [33]. A number of in vitro studies revealed that lanatoside C induced mitochondrial membrane potential loss and apoptosis via its action on protein kinase δ [37], induction of G2/M cell cycle arrest and inhibition of cancer cell growth by attenuating the MAPK, Wnt, JAK-STAT, and PI3K/AKT/mTOR signaling pathways [38]. Peruvoside is a novel Src inhibitor that causes cell cycle arrest in the G₂/M phase. It upregulates CDKN1A expression and activates the cleavage of caspases 3, 8 and PARP, leading to apoptosis. Therefore, peruvoside shows a strong anti-leukemic effect and may serve as a new anti-leukemia drug in the future [39]. In addition, cerberin causes significant G2/M cell cycle arrest by caspase 3/7 activation and PI3K/AKT/mTOR signaling inhibition [40]. Strophanthidin (SPTD), as a unique member of CGs without sugar rings, was also reported to inhibit the expression of several key kinases, e.g., MEK1, PI3K, AKT and GSK3α, downregulating MAPK and PI3K/AKT/mTOR signaling [12]. However, so far, few reports have investigated the mechanism of action of GCs on a proteome scale.

In the present work, by using MS-based quantitative proteomics analysis, we profiled the expression of the whole proteome in A549 lung cancer cells with and without SPTD treatment. Interestingly, we found that SPTD significantly raised the level of tumor necrosis factor (TNF)-related apoptosis-inducing ligand receptor 2 (TRAIL-R2), also known as death receptor 5 (DR5) in A549 cells. Inducing the apoptosis of tumor cells is one of the key strategies for the treatment of tumors [41]. There are two main signaling pathways that lead to apoptosis, namely endogenous pathways and exogenous pathways [42]. The exogenous apoptosis pathway is initiated by the ligand activation of death receptors [43]. The tumor necrosis factor (TNF)-related apoptosis-inducing ligand (TRAIL, also know Apo2L) is a member of the TNF superfamily which are the first death ligands identified for anticancer therapeutic targets [30,44]. However, the early stage of treatments based on TNF had serious side effects [45,46]. A few years later, it was reported that systemic therapy based on TRAIL-induced apoptosis in cancer cells killed tumor cells without cytotoxicity to normal cells. Thus, the development of TRAIL receptor (TRAIL-R) agonists (TRAs) as potential novel cancer therapies for clinical applications has been triggered [47,48,49].

TRAIL binds to death receptor 1 (TRAIL-R1), also known as DR4 or TNFRSF10A, or to death receptor 2 (TRAIL-R2), also known as DR5 or TNFRSF10B, to recruit a Fas-associated protein with a death domain (FADD) to form the death-inducing signaling complex (DISC) [30]. Then, FADD recruits pro-caspases 3, 6, or 8, activating the caspases via the auto-cleavage of the pro-caspases to trigger apoptosis [47,50,51]. An in vivo study on a nude mouse xenograft model with subcutaneous lung cancer showed that the knockdown or knockout of DR5 significantly increased lung cancer metastasis [52]. The study further demonstrated that when DR5 was suppressed, FADD and caspase 8 might recruit and stabilize TRAF2 to form metastatic and invasion signaling complexes, which in turn leads to the activation of ERK and JNK/AP-1 signaling to mediate the elevation/activation of matrix metalloproteinase-1 (MMP1), ultimately promoting cancer invasion and metastasis [52]. It has been reported that glioblastoma-derived stem cells (GSCs)-enriched neurospheres were resistant to the TRAIL-based treatment due to the low expression of DR4/5. However, the chemotherapeutic cisplatin could upregulate the level of DR5, restoring the TRAIL-induced apoptosis in the neurospheres [53]. The induced expression of TRAIL by retinoids [54] or histone deacetylase inhibitors (HDACIs) [55] in leukemia was also shown to cause blast apoptosis, contributing to the therapeutic efficiency of retinoids and HDACIs for leukemia [54,55]. Collectively, activating the TRAIL apoptotic pathway has been thought to be an effective way to kill cancer cells [43,47,50,56].

Interestingly, our study shows here that SPTD upregulated DR5 expression to activate caspases 3/6/8, in particular caspase 3, which in turn promoted the expression of downstream proteins apoptotic chromatin condensation inducer in the nucleus (ACIN1, FC = 1.44) and Prelamin-A/C (LMNA, FC = 1.75) in A549 cells. Both of the two downstream proteins can induce apoptosis through chromatin condensation [57]. ACIN1 (also known as Acinus), expressed in three isoforms, namely Acinus-L, Acinus-S, and Acinus-S′ [58], is a component of functional spliceosomes [59,60]. ACIN1 is cleaved and activated by caspase 2, inducing apoptotic chromatin condensation [58,61,62]. Lamin A/C (LMNA), presenting equally in the lamina of mammals, can be recruited by DNA repair proteins IFFO1 and XRCC4 to the DNA double-strand breaks to inhibit chromosome translocation via immobilizing broken DNA ends to induce chromatin condensation [57,63]. Our bioinformatics analysis also showed that most of the SPTD-induced DEPs, including ACIN1, interact directly or indirectly with each other via the core protein LMNA to be involved the TRAIL-induced apoptosis. This implies that the upregulation of ACIN1 and LMNA mediated by SPTD in A549 cells plays a crucial role in the anticancer action of SPTD. These results also imply that SPTD may cause DNA double-strand breaks, stimulating the interaction between Lamin A/C and IFFO1 or XRCC [63].

It is worth pointing out that the most upregulated protein, BANF1, by SPT is involved in the induction of apoptosis though interacting with LMNA. Banf1 is a non-specific DNA-binding protein, playing an important role in mitotic nuclear reassembly, chromatin organization and gene expression. It controls cellular DNA damage response towards oxidative stress by regulating poly [ADP-ribose] polymerase 1 (PARP1) [24,64]. In eukaryotic cells, the genome stored in a single nuclear compartment is divided into numerous chromosomes. If single chromosomes form a single nucleus (micronucleus), they are susceptible to DNA damage or even complete chromosome fragmentation. Being a common feature of cancer cells, the micronucleus is thought to be the main driving force of cancer genome evolution. Therefore, packaging all chromosomes into a single nucleus is essential for maintaining the integrity and health of the genome [23,24]. Samwer et al. revealed that BANF1 is a key factor in guiding membrane to the formation of a single nucleus, and that nuclear assembly relies on the ability of BANF1 to bridge distant DNA sites [23,24]. Our study here indicates that SPTD-induced BANF1 overexpression facilitated the chromosome condensation via interaction with LMNA, leading to apoptosis.

As mentioned before, it has been reported that SPTD reduced the expression of several key protein kinases, such as PI3K, AKT, MEK1, GSK3α and mTOR, inhibiting MAPK, PI3K/AKT/mTOR signaling [12]. In our present work, the quantification on the whole proteome did not detect pronounced changes in the level of these kinases described above. However, our bioinformatics analysis indicated that both of the upregulated and downregulated DEPs, including Rac GTPase-activating protein 1 (RACGAP1), Rho-related GTP-binding protein RhoB (RHOB), tumor necrosis factor receptor superfamily member 10B (TNFRSF10B/DR5), collagen alpha-1(V) chain (COL5A1), ataxin-10 (ATXN10), sequestosome-1 (SQSTM1) and Golgin subfamily A member 1 (GOLGA1), etc., interact with each other via TGFβ, VEGF and PDGF to inhibit ERK/MARK and PI3K/AKT signaling, being in consistence with the previous report [12]. The extracellular signal-regulated kinase 1/2 (ERK) belongs to the mitogen-activated protein kinase (MAPK) family and is responsible for delivering extracellular signals to intracellular targets. The MAPK cascade is a central signaling pathway that regulates a variety of cellular processes, e.g., proliferation, differentiation, apoptosis and stress response [65]. Therefore, the dysregulation or dysfunction of these cascades is associated with the induction and development of diseases such as cancer, diabetes, autoimmune diseases and dysplasia. The MAPK pathway includes three major kinases, MAPK, MAPK kinase (MAPKK) and MAPK kinase kinase (MPKKK), which phosphorylate and activate downstream proteins. A number of studies have shown that the overactivation of upstream proteins and kinases in the ERK/MAPK pathway induces a variety of diseases, including cancer, inflammation, developmental disorders and neurological disorders [66,67,68]. The Ras/Raf/MAPK (MEK)/ERK pathway is the most important signaling cascade of all of the MAPK signaling pathways and plays a vital role in the survival and development of tumor cells. The downstream of ERK activation plays a prominent role in tumor proliferation, invasion and metastasis, and the ERK/MAPK signaling pathway is closely related to tumor extracellular matrix degradation and tumor angiogenesis. A systematic review investigating the potential of Ras/Raf/MAPK(MEK)/ERK signaling pathway inhibitors in ovarian cancer showed a clinical benefit rate (CBR) of 63% and an overall response rate (ORR) of 13% in patients with ovarian cancer treated with MAPK inhibitors. Compared with other histological subtypes, MAPK inhibitors have higher efficacy in low-grade ovarian cancer and are tolerable and less toxic to patients [69]. With regard to this, our study here shows that SPTD is a potential inhibitor towards ERK/MAPK and PI3K/AKT signaling.

In the protein–protein interaction (PPI) network for the regulation of ERK/MAPK signaling, notably, there are three SPTD-downregulated proteins, PSMC3 (FC = −1.48), SQSTM1 (FC = −1.49) and COL5A1 (FC = −1.70), which are closely associated with the inhibition of ERK/MARK signaling. The proteasome 26S subunit ATPase (PSMC) family consists of six members: PSMC1–PSMC6, which partially constitute the formation of the 19S regulatory complex. This complex plays an important role in the regulation of the 26S proteasome, which in turn catalyzes substrate unfolding and translocation into the 20S proteasome [70,71]. There is also a positive correlation between PSMC family genes and ubiquinone metabolism, cell cycle and cytoskeletal remodeling. The results of high-throughput transcriptomic analysis showed that the expression profile of PSMC3 in breast cancer was significantly higher than that in normal breast tissue, being positively associated with low survival. Wang et al. analyzed caspase-associated apoptosis genes in gliomas by RNA-seq and bioinformatics and verified that PSMC3 is a key apoptosis gene involved in the regulation of mRNA metabolism in glioma cells [72]. The sequestosome-1 (SQSTM1) protein also plays an important role in the ubiquitin-proteasome system. The ubiquitin ligase RNF26 recruits and ubiquitinates the scaffold p62/sequestosome-1 (p62/SQSTM1) to attract the ubiquitin-binding domain (UBD) of a variety of vesicle adaptors. The resulting molecular bridge restricts homologous vesicles in the perinuclear region and organizes endosomal pathways to achieve efficient species transfer and ligand-induced signaling receptor clearance [73]. The vesicles can then be released from their perinuclear locations by RNF26-related deubiquitinases (DUBs) USP15 for rapid transport to the pericellular periphery, completing the dynamic cycle [73]. UPS and autophagy are two main intracellular degradation pathways, and p62 affects the activity of UPS and autophagy by bridging the ubiquitin-proteasome system and autophagy [74,75]. The recognition of ubiquitinated proteins during autophagy is mediated by the non-covalent interaction of the ubiquitin receptor with ubiquitin through its ubiquitin-binding domain, and p62/SQSMT1 is the first protein having this linker function [76]. One of the key roles of p62 is the delivery of various ubiquitinated proteins bound to its UBA domain to autophagosomes via the LIR domain, ultimately leading to their degradation by lysosomes [74,77]. SQSTM1 interacting with multiple binding chaperones enables p62 to act as a major regulator of the activation of Nrf2, mTORC1 and NF-κB signaling pathways, linking p62 to the oxidative defense system, nutrient sensing and inflammation and tumors, respectively [78,79]. Interestingly, the mechanism of action of the death receptors DR4 and DR5 also requires p62 to function optimally. Thus, the cellular activation of TRAIL promotes the polyubiquitination of caspase 3 through a cullin-8-dependent process [80]. Notably, p62 binds to polyubiquitinated caspase 8, and its recruitment to oligomeric spots is required to trigger the apoptotic pathway [80]. This suggests that the p62 spot is a signal transduction hub that can determine cell survival by triggering the TRAF6-NF-κB pathway, or by activating aggregation of caspase 8 and its downstream effector caspase. With regard to the opposite roles in cancer cells, p62/SQSTM1 is thought to be a Janus. It can serve a suppressor against tumorigenesis, but its accumulation was also found in various types of tumors [81]. Our data here revealed that SQSTM1 was downregulated by SPTD, most likely benefiting the anticancer capacity by facilitating the degradation of the key kinases such as ERK, MAPK and AKT.

Collagen is a major component of the tumor microenvironment, influencing the progression of cancer cells and being involved in chemoresistance [82]. Collagen deposition is a pathological feature in the tumor microenvironment [83]. Studies have shown that collagen, cancer cells and other components of the tumor microenvironment form a complex network of mutual reinforcement to promote the proliferation, migration and metastasis of cancer cells [84]. The three polypeptide chains α1(V), α2(V) and α3(V) encoded by the genes COL1A2, COL3A5 and COL1A5 make up the collagen V protein [85]. Among these, COL5A1 may be a new prognostic factor in patients with lung adenocarcinoma, and the miR-29b/COL5A1 axis can regulate bone metabolism and fibrosis response [86,87]. COL5A1 is highly expressed in ovarian cancer cells and tissues. A knockdown of COL5A1 inhibits the proliferation and migration of ovarian cancer cells. The study by Zhang et al. also showed that COL5A1 was overexpressed in paclitaxel-resistant ovarian cancer cells compared to their respective paclitaxel-sensitive cells. The elevated COL5A1 expression is associated with worse survival outcomes, thus COL5A1 is a potential biomarker predicting ovarian cancer progression and paclitaxel resistance [88]. In addition, COL5A1 was shown to be involved in extracellular remodeling and the regulation of actin filaments in glioblastoma (GBM) metastasis, and the COL5A1-PPRC1-ESM1 axis may represent a new therapeutic target for GBM [89]. In our present work, the SPTD-induced downregulation of COL5A1 was found to be involved in inhibiting p38 MARK/AKT/ERK signaling due to a weakening the interaction with PDGF and TGFβ.

Collectively, our data in the present work suggest that the SPTD-induced downregulation of PSMC3, SQSTM1 and COL5A1 contributes to the inhibition of the growth of A549 cells, perhaps by promoting the degradation of ubiquitinated protein kinases, e.g., ERK, MAPK and AKT, and by regulating the microenvironment of the cancer cells.

## 4. Materials and Methods

### 4.1. Materials

Strophanthidin (SPTD) was purchased from Yuanye Bio-Technology Co., Ltd. (B24384, HPLC ≥ 90%, Shanghai, China), and was dissolved in 100% DMSO to prepare its 10 mM stock solution. Human A549 non-small cell lung cancer cells and human HEK293T renal epithelial cells were purchased from the Institute of Basic Medicine at the Chinese Academy of Medical Sciences. Phosphate buffer saline (PBS), cell scrapers, cell petri dishes, centrifugal tubes and the RNase A of the DNA content detection kit were provided by Beijing Solaibao Technology Co., Ltd. (Beijing, China). PS (penicillin-streptomycin) were provided by Merck(Freiburg, Germany).The Total Protein Extraction Kit was obtained from BestBio (Beijing, China) and the Enhanced BCA Protein Assay Kit was purchased from Beyotime Biotechnology Co., Ltd. (Shanghai, China). The Cell Counting Kit-8 (CCK-8) was purchased from MedChem Express (Chiyoda, Japan), while the Apoptosis Detection Kit was obtained from BD Sciences (San Jose, CA, USA). Urea was purchased from Beijing Inokai Technology Co., Ltd. (Beijing, China), dithiothreitol (DTT), iodoacetamide (IAA) and trypsin from Sigma-Aldrich (St. Louis, MI, USA), and formic acid from Honeywell (Charlotte, NC, USA). Trifluoroacetic acid was purchased from Alfa Aesar (Tianjin, China), HEPES from Beijing Baotuoda Technology Co., Ltd. (Beijing, China) and hydroxylamine hydrochloric acid from Adamas Reagent Co., Ltd. (Shanghai, China). Chromatographic-grade water, mass spectrometric-grade acetonitrile and water, FBS (fetal bovine serum), tandem-mass-tag (TMT) labeling reagent, confocal culture dish and low adsorption tubes were purchased from Thermo Fisher Technology China Co., Ltd. (Waltham, MA, USA). The C18 cartridge and analytical column were purchased from Waters (Milford, MA, USA). Three antibodies for cleaved caspase 3 (#9664), cleaved caspase 6 (#9761) and cleaved caspase 8 (#98134), respectively, were purchased from Cell Signaling Technology (Danvers, MA, USA), the secondary antibody (ab150073) was purchased from Abcam (Cambridge, MA, USA). Ultrapure water was produced by Millipore pure water system.

### 4.2. Cell Culture

A549 and 293T cells were cultured in DMEM culture medium (Dulbecco’s Modified Eagle’s Medium, Gibco) containing 10% FBS, (Gibco, Thermo Fisher Scientific, Waltham, MA, USA) and 1% PS (GE Heathcare Life Sciences, Chicago, IL, USA). All cells were cultured in 5% CO_2_ in a 37 °C incubator.

### 4.3. Cell Viability Assay

The A549 or HEK293T cells were transferred into a 96-well plate at a density of 6000 cells/well and cultured in DMEM for 24 h at 37 °C. The cells were then incubated with various concentrations of SPTD (0.01 μM–20 μM), which were stepwise diluted from 10 mM stock solution by cell media containing 0.2% DMSO, for 24 h. Thereafter, 10 μL of CCK-8 reagent was added into each well for 2 h of reaction and the optical absorption density (OD) of each well was measured at 450 nm using a microplate reader (TECAN F50, Männedorf, Switzerland).

### 4.4. Apoptosis Assay

A549 cells, cultured in medium containing 1.0 μM SPTD and 0.2% DMSO for 24 h, were collected by trypsin digestion without EDTA. An Annexin V-FITC/PI apoptosis double staining kit was used to detect the effect of SPTD on the apoptosis of A549 cells. A small number of cells were stained with fluorescein isothiocyanate (FTIC) and PI as controls, and the remaining cells were stained with both FITC and PI for a formal test. Apoptosis assays were performed on a Calibur flow cytometer (BD, Franklin Lakes, NJ, USA). The data were quantified by FlowJo software ver. 7.6.1, Engine 2.79000, Java Ver.14.1-b02 (Treestar, St. Chico, CA, USA).

### 4.5. Cell Cycle Assay

Similar to the cell viability assay, A549 cells were cultured in medium containing 1.0 μM SPTD and 0.2% DMSO for 24 h. The cells were harvested by trypsin digestion and washed with 4 °C PBS, and then cells were fixed with 78% ethanol at −20 °C overnight. The cells were resuspended with RNase A of the DNA content detection kit for 30 min at 37 °C. Then, the cells were incubated with propidium iodide (PI) for 30 min at 4 °C in the dark. The samples were detected on a Calibur flow cytometer and recorded at an excitation wavelength of 488 nm.

### 4.6. Protein Extraction

A549 cells were cultured in medium containing 1.0 μM SPTD and 0.2% DMSO for 24 h, and scraped from the dish for lysis with cell lysis buffer. Then protease inhibitor and phosphatase inhibitor were added, shocking at 4 °C for 90 min. The mixed lysate was centrifuged for 20 min at 8000× *g*, followed by the collection of the supernatants. The protein concentration of the supernatants was determined by BCA Kit. A549 cells were cultured in medium without SPTD and extracted proteins following the same procedure as the control samples of subsequent quantitative proteomics analysis. The entire quantitative proteomics analysis was carried out in three independent biological replicates.

### 4.7. Protein Digestion

An aliquot (300 μg) of the protein samples derived from control and SPTD-treated A549 cells was individually added with urea at a final concentration of 8 M in a low protein binding microcentrifuge tube, and DTT was added to obtain a final concentration in the solution of 5 mM for incubation at 25 °C for 30 min. An appropriate amount of IAA was added to each sample at a final concentration of 10 mM for reaction at 25 °C for 1 h. After that, the protein solutions were transferred to a 10 KDa ultrafiltration tube and centrifuged at 8000× *g* for 30 min. Thereafter, the sample was washed twice with 300 μL 50 mM TEAB solution; 200 μL of 50 mM TEAB and 70 μL pancreatin were added to the ultrafiltration tube. The mixture was shocked at 25 °C for 16 h. The digested protein sample was centrifuged at 10,000× *g* in a 10 KDa ultrafiltration tube for 40 min, followed by washing twice with 20 μL of 50 mM TEAB and 30% acetonitrile. The obtained tryptic digest was dried by vacuum centrifuge (CentriVap, ThermoFisher Scientific, Waltham, MA, USA), and then redissolved and diluted to 100 μg/mL with 50 mM HEPES (pH 8.5) for subsequent TMT labeling.

### 4.8. TMT Labeling

An aliquot (41 μL) of 100% acetonitrile was added to 0.8 mg TMT powder which had been restored to room temperature, then 100 μL of TMT solution was added to each tryptic peptide sample described above, and the mixture was allowed to react at 25 °C with shaking for 1 h and then quenched by adding 8 μL of 5% hydroxylamine. Three biological replicates of controls were labeled with 126, 127C and 127N labels, respectively, and the samples of SPTD treated samples were labeled with 128C, 128N and 131 labels, respectively. The labeled peptides derived from control and SPTD treatment groups and were equivalently mixed and dried by vacuum dryer, desalted and dried again as described previously [20].

The labeled peptide mixture was re-dissolved in 100 μL of 2% (vol/vol) ACN/H2O containing 4.5 mM ammonium formate (pH 10) for basic reverse-phase chromatography pre-fractionation by HPLC (Agilent Technologies 1260 infinity, Santa Clara, CA, USA) with an Agilent ZORBAX 300 Extend-C18 column. The gradient started with 0% phase B (90% (vol/vol) acetonitrile containing 4.5 mM ammonium formate, pH = 10) until 7 min and continuously increased to 16% B at 13 min, 40% B at 73 min, 44% B at 77 min and 60% B at 82 min until 96 min, then increased to 90% B at 100 min. The flow rate was 1 mL/min. The fractions were collected chronologically into twelve tubes from 3–96 min on the basis of crossfading fractionation [90]. The pooled fractions were spun until dried and re-dissolved in H_2_O containing 0.1% FA to a 500 μg/μL concentration of peptides for mass spectrometry analysis.

### 4.9. NanoLC-MS/MS Analysis

The mass spectrometric quantitative analysis of 1 μL of the re-dissolved TMT-labeled peptide fractions was performed on an Orbitrap Fusion Lumos mass spectrometer coupled with an EASY-nLC 1200 nano-UPLC system equipped with an Acclaim™ PepMap™ 100 pre-column (20 mm × 75 μm, 3 μm) and an Acclaim™ PepMap™ RSLC C18 analytical column (150 mm × 75 μm, 2 μm) (Thermo Fisher Scientific, Waltham, MA, USA). The UPLC gradient started with 2% B and increased to 7% at 7 min, then to 20% at 69 min, 35% at 90 min and sharply to 95% within 5 min, remained for 4 min, and finally decreased to 2% within 8 min and remained for 3 min.

The voltage of electrospray ionization (ESI) was set as 2200 V, the ion transfer tube temperature was 320 °C. For primary mass spectrometry analysis, an Orbitrap detector was operated with a mass resolution of 120,000 and a scanning range from 350 to 1700 *m*/*z*. The Orbitrap was also used as the detector of MS/MS with a mass resolution of 15,000, and of MS^3^ with a resolution of 50,000 and a scanning range of 100–200 *m*/*z*. The HCD fragmentation cell ran with a collision energy of 23% for the second mass spectrometry (MS/MS) analysis, and with a collision energy of 60% for the third mass spectrometry (MS^3^) analysis.

### 4.10. Protein Database Search

The protein database search was performed using Proteome Discoverer 2.5 (Thermo Fisher Scientific, Waltham, MA, USA) with the search engine Sequest HT for peptide spectrum matching (PSM). The oxidation of methionine and the phosphorylation of serine, threonine and tyrosine were selected as dynamic modifications. The carbamidomethylation at cysteine and TMT labeling at the N-terminus of any n-peptide were selected as static modifications. The quantitative results were normalized based on the total peptide amount in each sample. Only proteins identified with a false discovery rate (FDR) ≤ 0.01, *p*-value ≤ 0.05 and abundance ratio (treated vs. control) ≤ 0.77 or ≥1.30 were included for bioinformatics analysis.

### 4.11. Bioinformatics Analysis

STRING (Ver. 11.5, https://cn.string-db.org on 12 January 2023) and Ingenuity Pathway Analysis (IPA) (QIAGEN Digital Insights) programs were used to perform bioinformatics analysis. Only proteins identified with a false discovery rate (FDR) ≤ 0.01, *p*-value ≤ 0.05 and abundance ratio (treated vs. control) ≤ 0.77 or ≥1.30 were identified as differentially expressed proteins (DEPs) and included for bioinformatics analysis.

### 4.12. Immunofluorescence

Two groups of A549 cells were individually cultured with DMEM in confocal culture dishes for 24 h. One group of cells was cultured in the absence of SPTD, another in the presence of 1.0 μM SPTD. Each group of cells was then fixed with methanol and acetic acid in a 3:1 (v/v) ratio for 10 min at −20 °C. The cells were then permeabilized in 0.1% Triton/PBS for 30 min and blocked with 5% BSA for 1 h. Thereafter, the primary antibodies for cleaved caspase 3, 6 and 8 were added to the cells and incubated at 4 °C overnight, and incubated for a further 1 h in the dark with the addition of secondary antibody (ab150073). Finally, the cells were incubated with 0.1 g/mL DAPI solution for 10 min, and fluorescence images were recorded by confocal laser scanning microscopy (OLYMPUS, FV3000, Tokyo, Japan). The excitation and emission wavelengths for the secondary antibody and for DAPI were 488 nm and 360 nm, and 525 nm and 460 nm, respectively.

## 5. Conclusions

By using mass spectrometry-based quantitative proteomics analysis, we discovered that strophanthidin (SPTD) upregulated 24 proteins, including Banf1 and LAMN, and downregulated 35 proteins, such as SRSF3 and COL1A5, in A549 cancer cells. Bioinformatics analysis revealed that the SPTD-induced differentially expressed proteins (DEPs) are closely associated with the death receptor signaling and ERK/MAPK signaling pathway. Importantly, we found that SPTD promoted the expression of tumor necrosis factor (TNF)-related apoptosis-inducing ligand receptor 2 (DR5) in A549 cells, which in turn activated caspase 3/6/8. The activated caspases consequently upregulated two downstream proteins, apoptotic chromatin condensation inducer in the nucleus (ACIN1) and prelamin-A/C (LMNA), both of which promoted apoptosis via chromatin condensation. Moreover, the SPTD-induced DEPs interacted with each other to downregulate the p38 MAPK/ERK signaling by promoting the degradation of ubiquitinated protein kinases, contributing to the SPTD inhibition of the growth of A549 cells. Furthermore, the collagen COL1A5 was also downregulated by SPTD, being favorable to the SPTD inhibition of the growth of A549 cells by modulating the cell microenvironment. These findings provide novel insights into better understanding the molecular mechanism of action of SPTD as a promising anticancer drug derived from natural resources.

## Data Availability

Data are contained in the article and Supplementary Materials.

## Supplementary Materials

The following supporting information can be downloaded at: https://www.mdpi.com/article/10.3390/molecules29040877/s1.
