# Supplementary material for: Strophanthidin Induces Apoptosis of Human Lung Adenocarcinoma Cells by Promoting TRAIL-DR5 Signaling
Source: Molecules. 2024 Feb 16;29(4):877. doi: 10.3390/molecules29040877 (PMC10892344; doi:10.3390/molecules29040877)
Supplement: Supplementary file 1 [file molecules-29-00877-s001.zip › molecules-2853623-Figures S1-S12.pdf]

Strophanthidin induces apoptosis of human lung adenocarcinoma cells by promoting TRAIL-DR5 signaling

Xiao Tian [1,2,+], Liangzhen Gu [2,3,+], Fangang Zeng[4], Xingkai Liu[2], Yang Zhou [2, 3], Yang Dou[2,3], Juanjuan Han[2], Yao Zhao[2,3], Yanyan Zhang[2], Qun Luo[*2,3] and Fuyi Wang[*1,2,3]

Supplementary Materials

Figure S1 – S12

Table S1 – S2

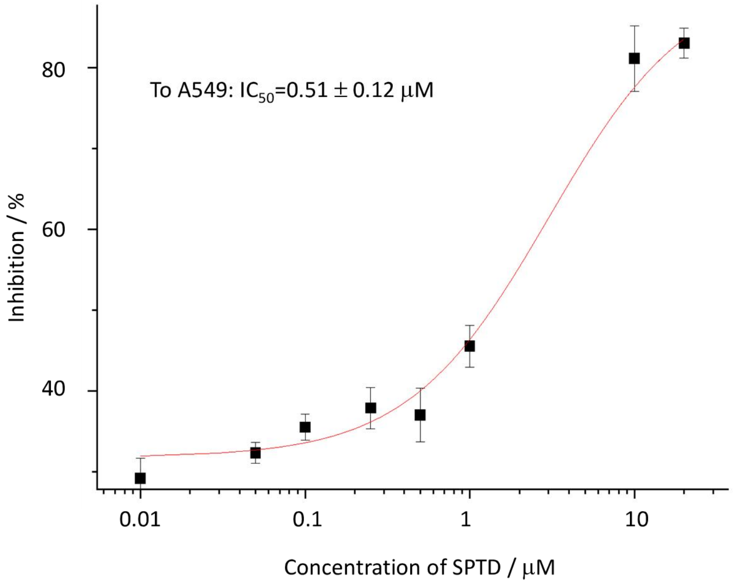

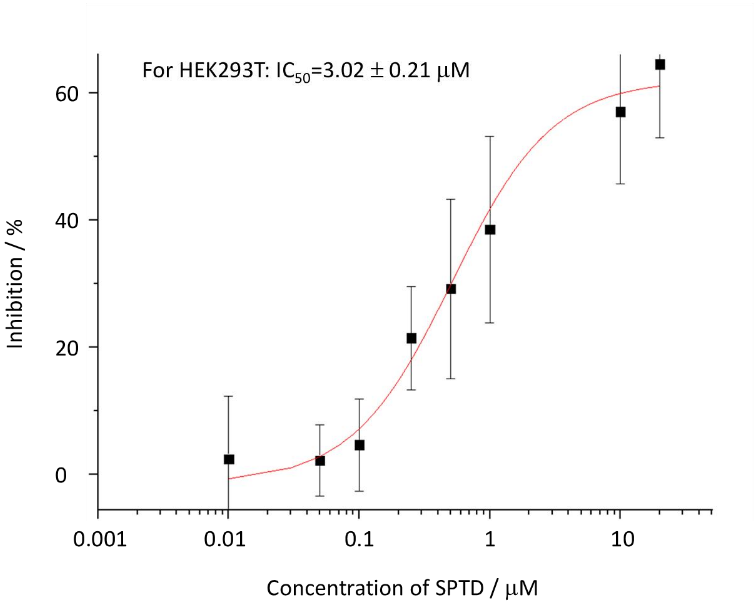

Figure S1. The inhibition of Strophanthidin (SPTD) on the growth of (top) A549 cells and (bottom) HEK293T cells incubated with various concentrations of SPTD at 37 °C for 24 h. The inhibition rate (%) are present as mean ± SD, n=6.

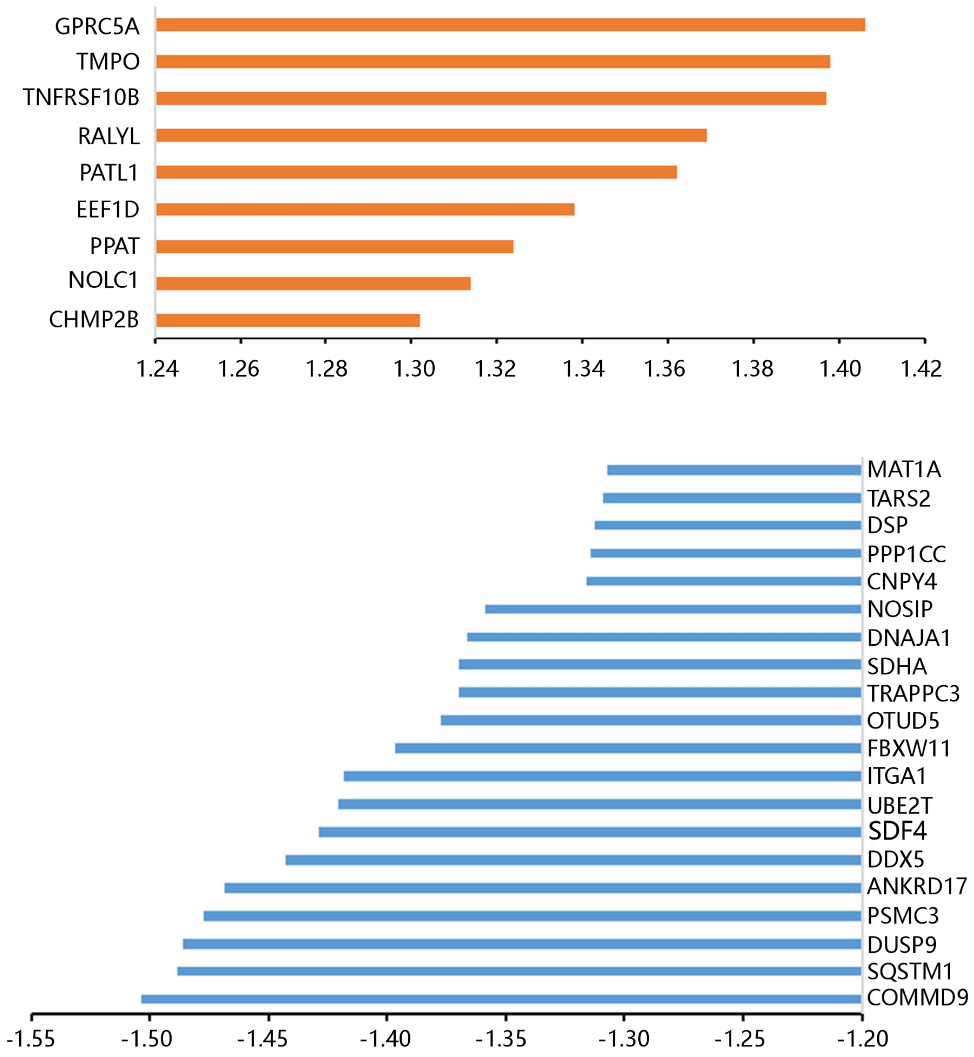

Figure S2. Differentially expressed proteins (DEPs) with a fold change |FC| ≥ 1.3 identified in A549 cells incubated with 1.0 μM Strophanthidin (SPTD) at 37 °C for 24 h, compared to those in A549 cells without treatment.

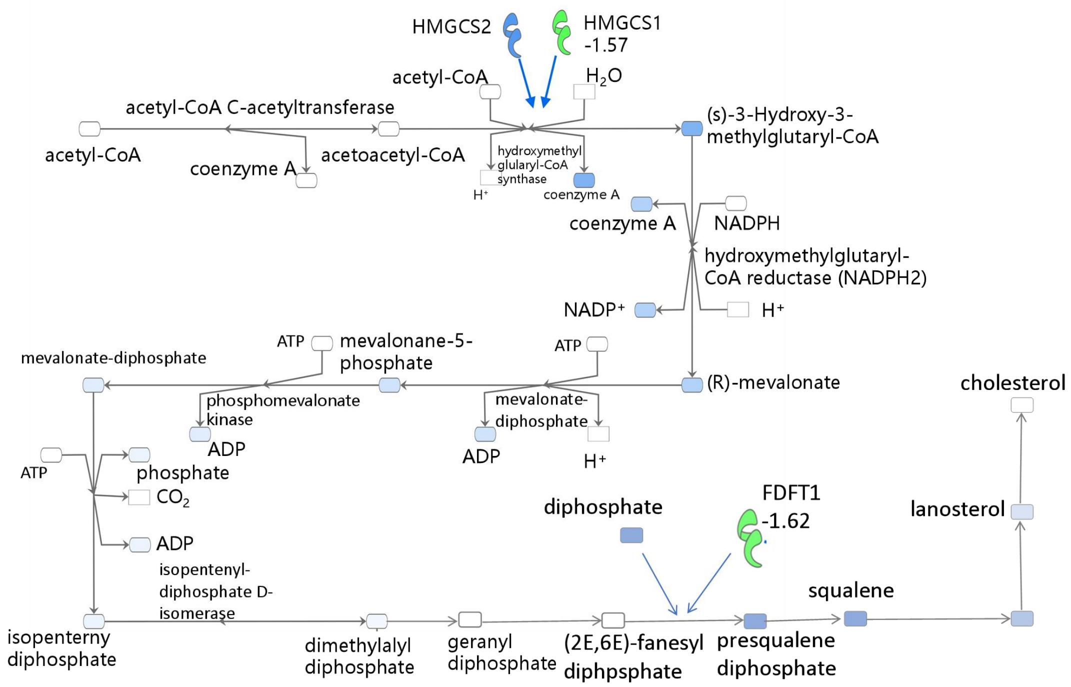

Figure S3. Schematic diagram super-pathway of cholesterol biosynthesis signaling pathway with which the DEPs with |FC| ≥1.3 identified in A549 cells incubated with 1.0 µM strophanthidin (SPTD) at 37 °C for 24 h, compared to those in A549 cells without treatment. The red color represents the protein (or complex) upregulated, green downregulated; the orange color represents the protein (or complex) predicted to be upregulated, blue to be downregulated.

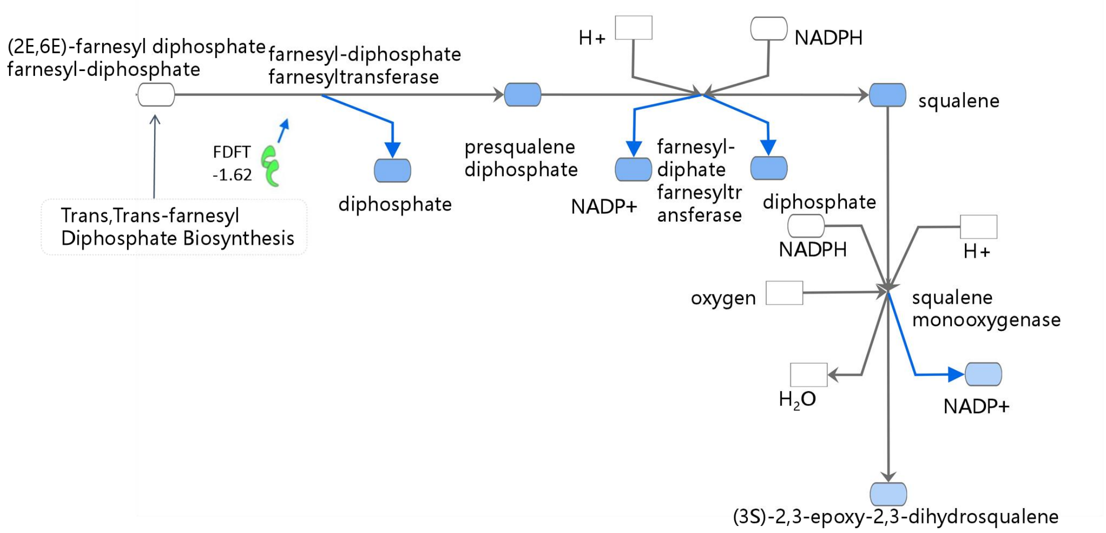

Figure S4. Schematic diagram super-pathway of epoxy-squalene biosynthesis signaling pathway with which the DEPs with |FC| ≥1.3 identified in A549 cells incubated with 1.0 μM SPTD at 37 °C for 24 h, compared to those in A549 cells without treatment. The red color represents the protein (or complex) upregulated, green downregulated; the orange color represents the protein (or complex) predicted to be upregulated, blue to be downregulated.

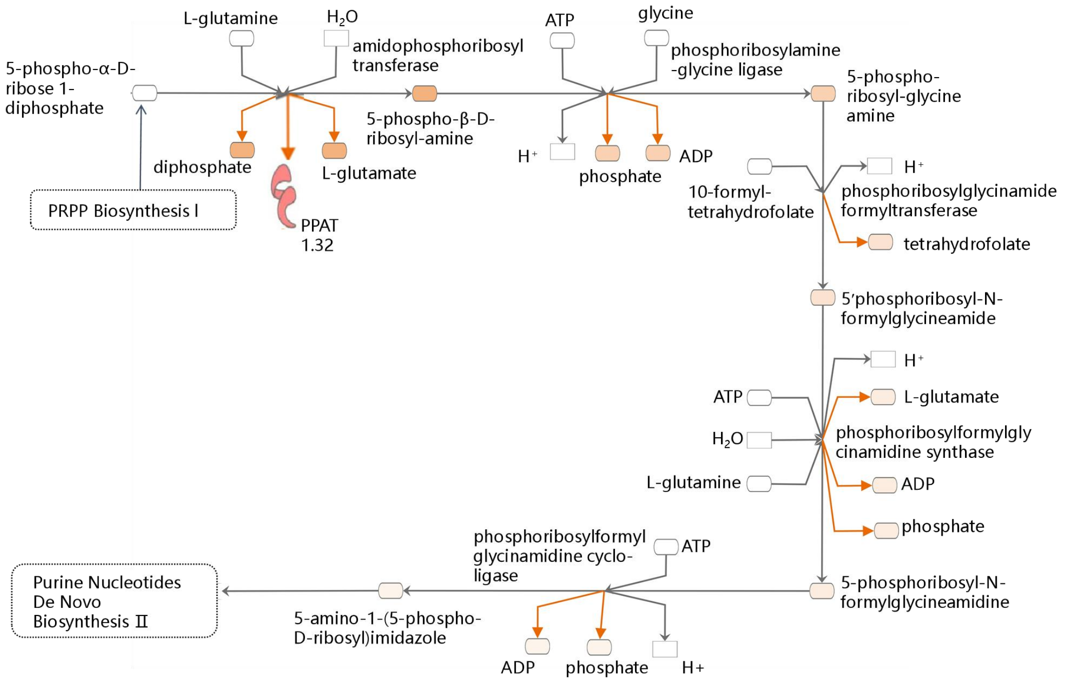

Figure S5. Schematic diagram super-pathway of 5-aminoimidazole ribonucleotide biosynthesis I signaling pathway with which the DEPs with |FC| ≥1.3 identified in A549 cells incubated with 1.0 μM SPTD at 37 °C for 24 h, compared to those in A549 cells without treatment. The red color represents the protein (or complex) upregulated, green downregulated; the orange color represents the protein (or complex) predicted to be upregulated, blue to be downregulated.

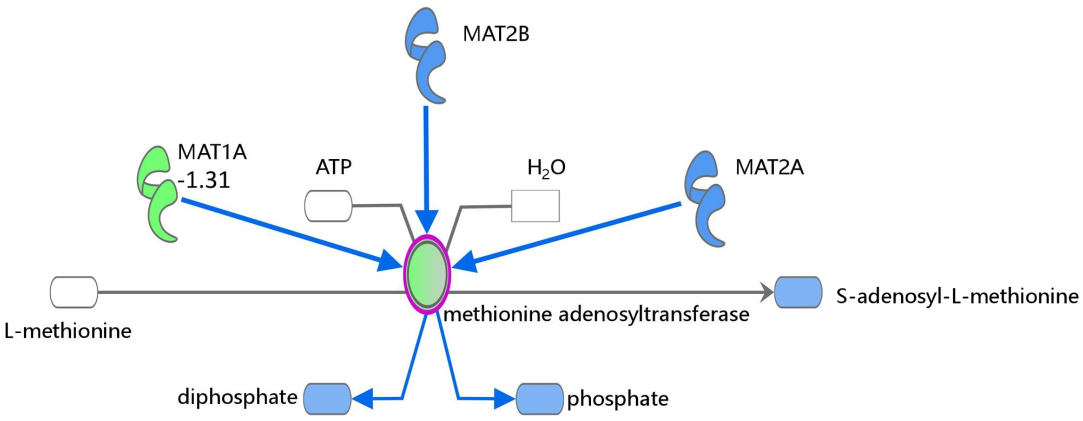

Figure S6. Schematic diagram super-pathway of S-adenosyl-L-methionine biosynthesis signaling pathway with which the DEPs with |FC| ≥1.3 identified in A549 cells incubated with 1.0 μM SPTD at 37 °C for 24 h, compared to those in A549 cells without treatment. The red color represents the protein (or complex) upregulated, green downregulated; the orange color represents the protein (or complex) predicted to be upregulated, blue to be downregulated.

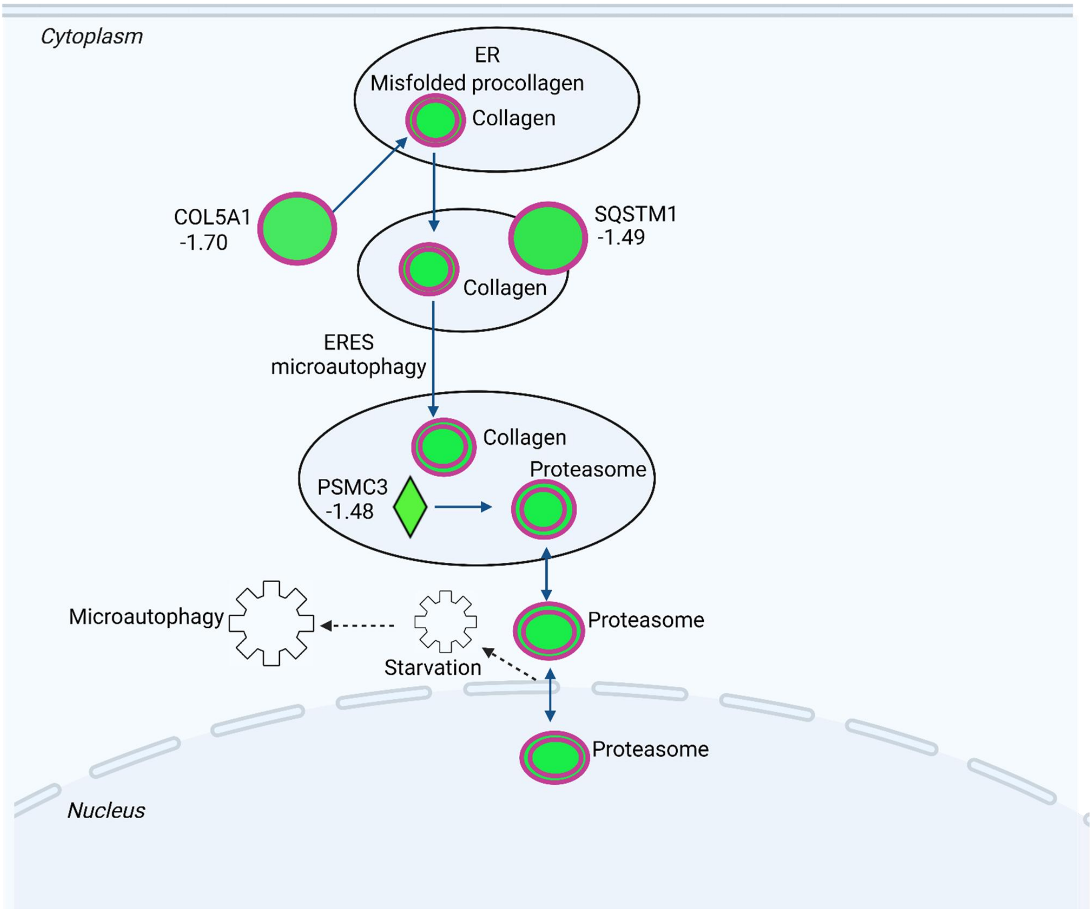

Figure S7. Schematic diagram super-pathway of micro-autophagy signaling pathway with which the DEPs with |FC| ≥1.3 identified in A549 cells incubated with 1.0 μM SPTD at 37 °C for 24 h, compared to those in A549 cells without treatment. The red color represents the protein (or complex) upregulated, green downregulated; the orange color represents the protein (or complex) predicted to be upregulated, blue to be downregulated.

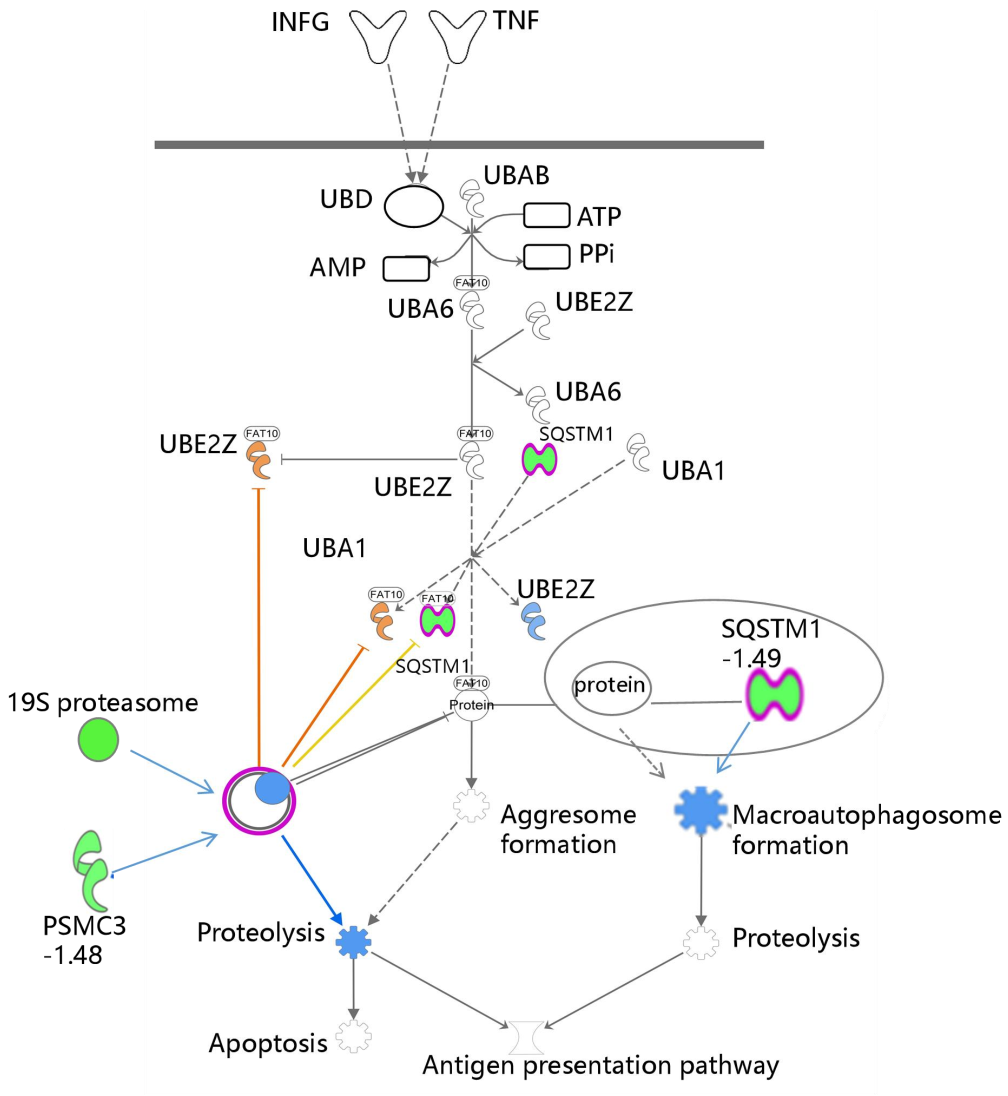

Figure S8. Schematic diagram super-pathway of FAT10 signaling pathway with which the DEPs with |FC| ≥1.3 identified in A549 cells incubated with 1.0 μM SPTD at 37 °C for 24 h, compared to those in A549 cells without treatment. The red color represents the protein (or complex) upregulated, green downregulated; the orange color represents the protein (or complex) predicted to be upregulated, blue to be downregulated.

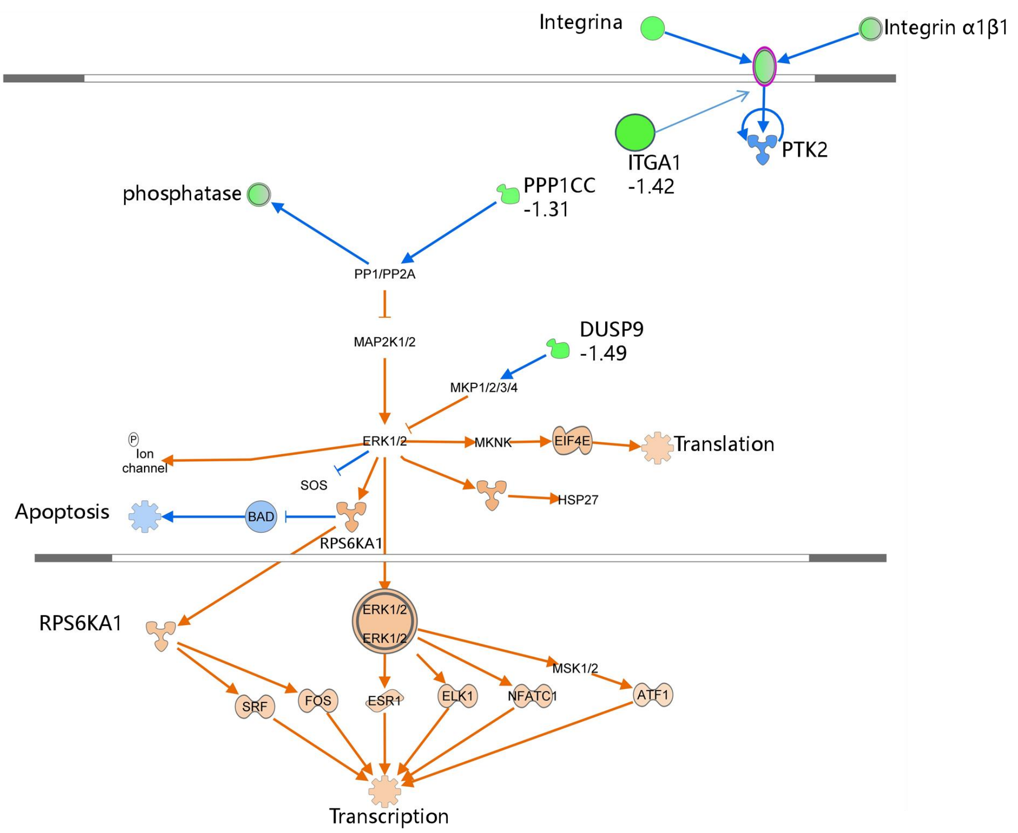

Figure S9. Schematic diagram super-pathway of ERK/MAPK signaling pathway with which the DEPs with |FC| ≥1.3 identified in A549 cells incubated with 1.0 μM SPTD at 37 °C for 24 h, compared to those in A549 cells without treatment. The red color represents the protein (or complex) upregulated, green downregulated; the orange color represents the protein (or complex) predicted to be upregulated, blue to be downregulated.

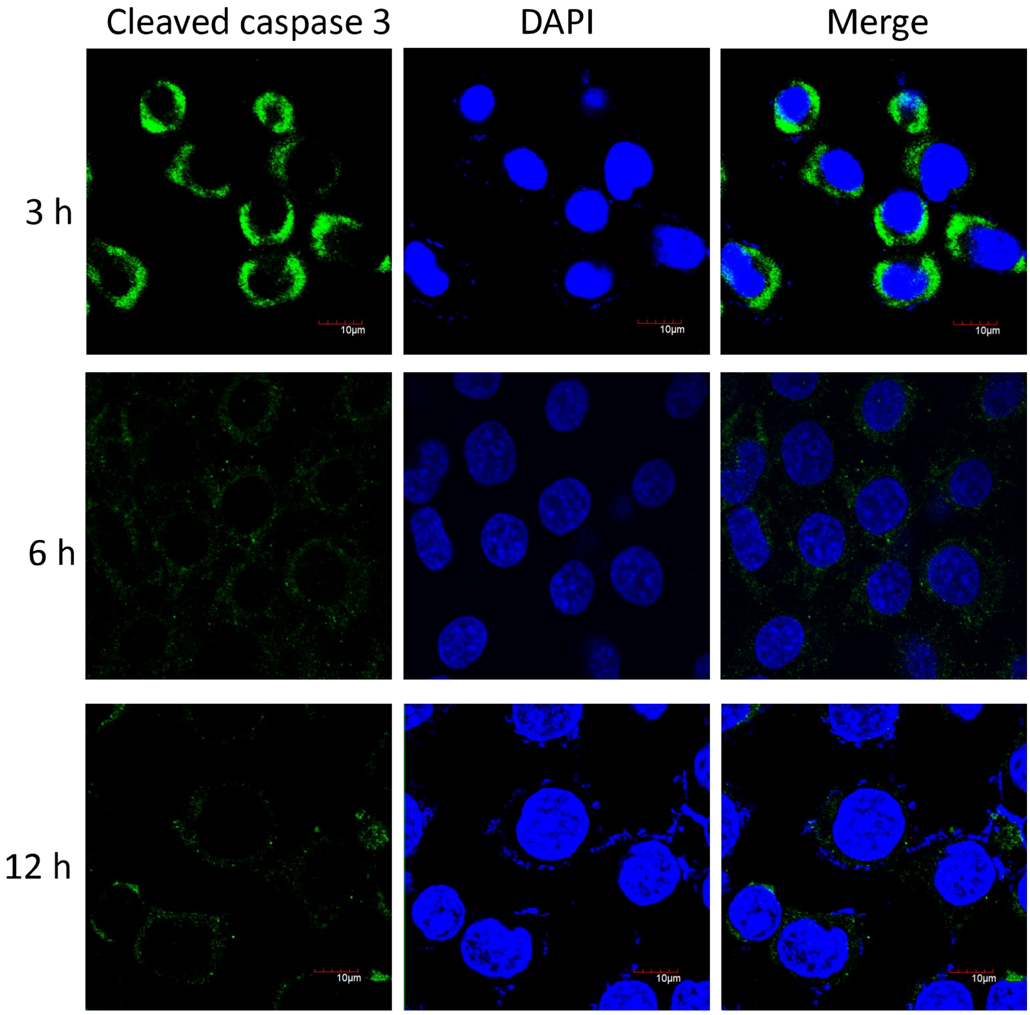

Figure S10. Immunofluorescence images of cleaved/activated caspase-3 in A549 cells treated with 1.0 μM SPTD for different times at 37 °C. Green fluorescence represents the target protein and blue fluorescence represents the nucleus strained by DAPI.

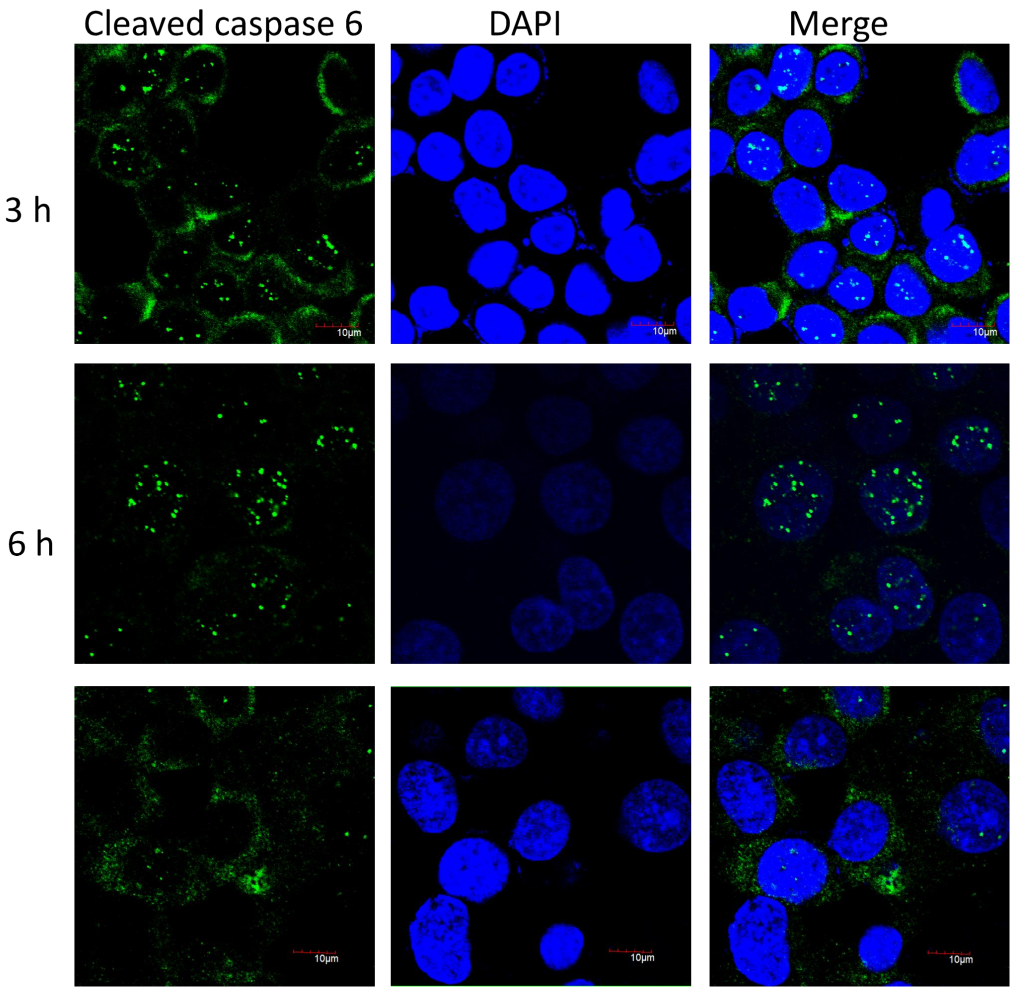

Figure S11. Immunofluorescence images of cleaved/activated caspase-6 in A549 cells treated with 1.0 μM SPTD for different times at 37 °C. Green fluorescence represents the target protein and blue fluorescence represents the nucleus strained by DAPI.

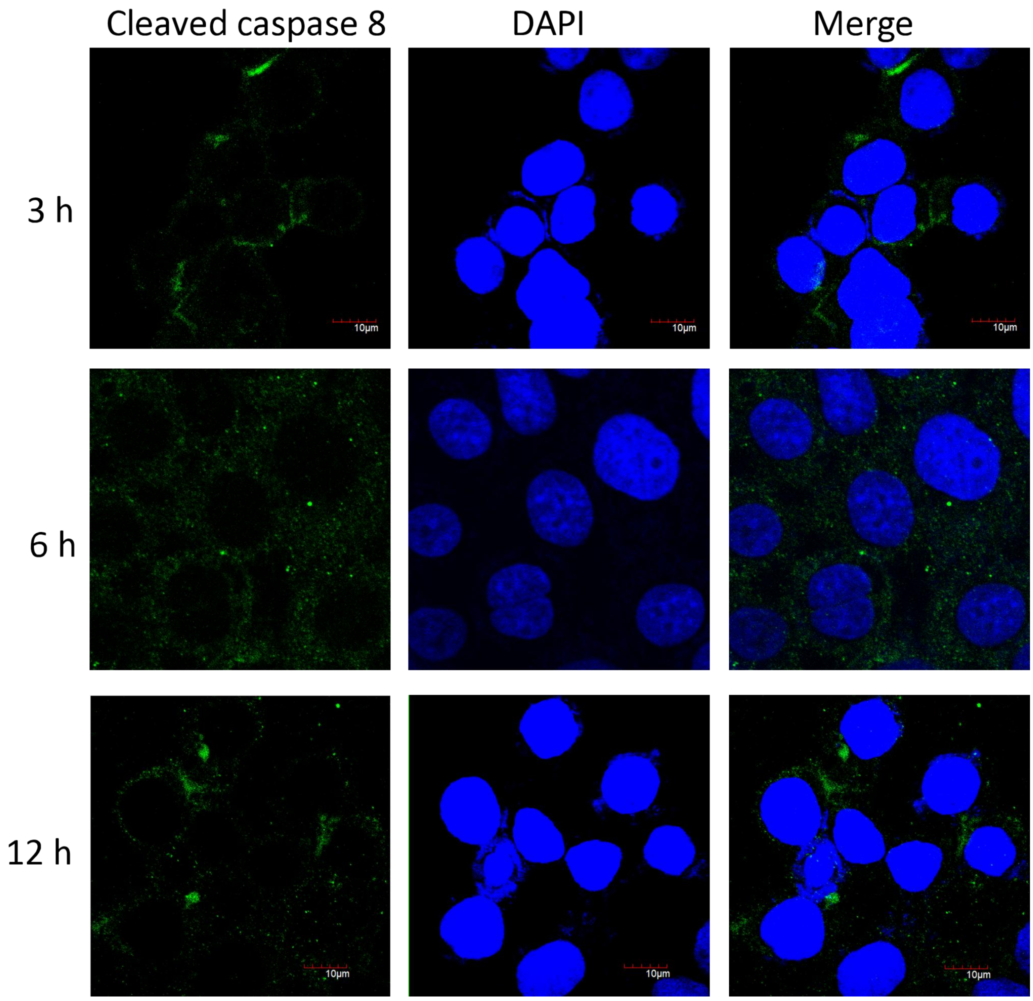

Figure S12. Immunofluorescence images of cleaved/activated caspase-8 in A549 cells treated with 1.0 μM SPTD for different times at 37 °C. Green fluorescence represents the target protein and blue fluorescence represents the nucleus strained by DAPI.
